# Expression and function of *nr4a2*, *lmx1b*, and *pitx3 *in zebrafish dopaminergic and noradrenergic neuronal development

**DOI:** 10.1186/1471-213X-7-135

**Published:** 2007-12-05

**Authors:** Alida Filippi, Katrin Dürr, Soojin Ryu, Marc Willaredt, Jochen Holzschuh, Wolfgang Driever

**Affiliations:** 1 Developmental Biology Department, Institute of Biology I, University of Freiburg, Hauptstrasse 1, D-79104 Freiburg, Germany; 2 Department of Neuroscience, Karolinska Institute, Retzius väg 8, 17177 Stockholm, Sweden; 3 IZN, University of Heidelberg, Im Neuenheimer Feld 307, D-69120 Heidelberg, Germany

## Abstract

**Background:**

Dopaminergic neurons form in diverse areas of the vertebrate di- and mesencephalon to constitute several major neuromodulatory systems. While much is known about mammalian mesencephalic dopaminergic neuron development, little is known about the specification of the diencephalic dopaminergic groups. The transcription factors Pitx3 and Lmx1b play an important role in mammalian mesencephalic dopaminergic specification, and Nurr1/Nr4a2 has been shown to contribute to specification of the dopaminergic neurotransmitter phenotype. We use zebrafish to analyze potentially evolutionarily conserved roles of these transcription factors in a vertebrate brain that lacks a mesencephalic dopaminergic system, but has an ascending dopaminergic system in the ventral diencephalon.

**Results:**

We use a combination of fluorescent *in situ *hybridization and immunohistochemistry to determine whether *nr4a2*, *lmx1b*, and *pitx3 *genes are expressed in mature dopaminergic neurons or in potential precursor populations. We identify a second *nr4a2 *paralogue, *nr4a2a*, and find it co-expressed with Tyrosine hydroxylase in preoptic, pretectal and retinal amacrine dopaminergic neurons, while *nr4a2b *is only expressed in preoptic and retinal dopaminergic neurons. Both zebrafish *nr4a2 *paralogues are not expressed in ventral diencephalic dopaminergic neurons with ascending projections. Combined morpholino antisense oligo mediated knock-down of both *nr4a2a *and *nr4a2b *transcripts reveals that all zebrafish dopaminergic neurons expressing *nr4a2a *depend on Nr4a2 activity for *tyrosine hydroxylase *and *dopamine transporter *expression. Zebrafish *lmx1b.1 *is expressed in noradrenergic neurons of the locus coeruleus and medulla oblongata, but knock-down reveals that it is specifically required for *tyrosine hydroxylase *expression only in the medulla oblongata area postrema noradrenergic neurons. Both *lmx1b *genes and *pitx3 *are not expressed in dopaminergic neurons, but in a diencephalic territory that might contain precursor cells for ventral diencephalic dopaminergic neurons. Upon morpholino knock-down of both *lmx1b *paralogues, the number of neurons in diencephalic dopaminergic clusters with ascending projections appears specifically reduced. Thus *lmx1b *paralogues may contribute to the generation of diencephalic dopaminergic precursors. Conversely, knock-down of *pitx3 *does not specifically affect any diencephalic DA cluster.

**Conclusion:**

Our data indicate a conserved evolutionary role of Nr4a2 proteins in specification of the neurotransmitter phenotype, albeit it appears to be only one of several regulatory modules of dopaminergic differentiation, as most ventral diencephalic dopaminergic neurons do not express *nr4a2 *genes in zebrafish. For zebrafish *lmx1b *genes, which are not expressed in mature dopaminergic neurons, our data suggest a role in diencephalic precursor populations contributing to the ascending dopaminergic systems. A di-mesencephalic longitudinal domain of *lmx1b *expression may be the basis for the expansion and posterior shift of ventral di-/mesencephalic dopaminergic populations with ascending projections during evolution.

## Background

Dopaminergic (DA) neurons control functions as diverse as initiation of movement, reward associated behaviours and hormonal homeostasis. Malfunction of DA input accordingly has been implicated in a broad range of diseases, including Parkinson's disease, schizophrenia, sleep disorders, and drug addiction [1]. In the mammalian brain, groups of DA neurons appear at stereotypic positions along the rostrocaudal axis, with defined projection patterns [2-5]. In the telencephalon, a small population of DA neurons develops in the olfactory bulb (group A16), where it makes locally restricted connections. Diencephalic DA neurons are subdivided into 5 groups (A11–A15), predominantly located in the hypothalamus except for groups A11, which extends into the thalamus, and A13, which develops in the zona incerta of the ventral thalamus. Projections from these groups are highly diverse, ranging from local projections in the hypothalamus (A12) to some of the most far-reaching DA projections of the diencephalospinal system (A11). The best characterized DA neurons are located in the ventral mesencephalon (mesDA), and classified into three different groups: in the retrorubral field (RrF, group A8), in the substantia nigra pars compacta (SNc, group A9) and in the ventral tegmental area (VTA, group A10). The A9 and A10 groups provide ascending projections establishing the nigrostriatal, the mesolimbic, and the mesocortical systems.

Due to their involvement in Parkinson's disease, the mesDA neurons have been the object of intensive studies aimed at identifying extrinsic and intrinsic molecules required for their specification and differentiation. In mice, the generation of mesDA progenitors requires Shh signaling from the floor plate and FGF8 signalling from the mid-hindbrain boundary organizer [6]. In combination with other patterning signals, a temporal sequence of transcription factor expression is established to control mesDA development (reviewed by [7]). Otx2, Lmx1b and En1/2 start to be expressed in the ventral midbrain at E9.0, shortly before Lmx1a and Msx1 (E9.5) [8-11]. Later, Ngn2 and Mash1 expression has been postulated to induce the conversion of floor-plate cells into neuronal progenitor cells [12,13]. When mesDA progenitors exit the cell cycle and start to differentiate, they initiate the expression of Nurr1/Nr4a2 [14], an orphan nuclear receptor belonging to the superfamily of ligand-activated transcription factors. Although Nurr1/Nr4a2 is expressed in other areas of the mouse embryonic brain, only mesDA groups have been reported to be affected in *Nurr1/Nr4a2 *mutants so far. In these mutants, the mesDA progenitors are normally formed but are lost at later stages of development [15,16]. Thus, Nurr1/Nr4a2 does not seem to be required for the formation of mDA progenitors, but it is necessary for their differentiation and maintenance. It has been postulated that Nurr1/Nr4a2 directly binds the *tyrosine hydroxylase *promoter to activate its expression [17], but the proposed mechanisms of Nurr1/Nr4a2 action have been challenged based on studies of the human *th *promoter [18]. The paired-like homeodomain transcription factor Pitx3 appears to control an independent late step leading to the maturation of mesDA neurons. In the mes-diencephalic area, Pitx3 expression at E12 overlaps with TH expression, and in adult mice is restricted to all mesDA neurons [19-21]. *Pitx3-/- *mutant mice appear to form mesDA precursors, but the final steps in differentiation, including expression of TH, and maintenance of mesDA precursors are deficient.

The control of diencephalic DA development is less well understood. Progenitors of the diencephalic group A13 in mouse express both Dlx1/2 and Pax6, but only Dlx1/2 plays a major role in the specification of these neurons [22]. For tuberal hypothalamic DA neurons in chick embryos Shh and Bmp7 from the prechordal mesoderm cooperate to induce the hypothalamic regional markers Nkx2.1 and Msx1/2, and dopaminergic differentiation in a Six3-dependent manner [23]. Recently, Otp has been identified as specifically required for development of the A11 DA group in mice [24], and for DA neurons of the posterior tuberculum and hypothalamus of zebrafish [24,25].

The roles of Otp in mice and zebrafish reveal that some mechanisms of DA neuronal specification appear to be conserved throughout evolution. However, from fish to mammals, the dopaminergic systems have evolved significantly, culminating in the observation that teleost fish do not develop dopaminergic neurons in the midbrain, but only in the forebrain [26-30]. Zebrafish DA neurons have been detected in the olfactory bulb (like mammalian A16), the subpallium (in mammals transient TH expression in lateral ganglionic eminence [2]), the preoptic region (mammalian A15; [3]), and in the pretectum (where TH expression is transient in mammalian embryos). Further, several clusters of DA neurons form in the ventral thalamus and hypothalamus. These clusters have been numbered [28], because in the embryo the anatomical correlates are not fully resolved for all groups: group 1 in the ventral thalamus, groups 2, 3, 4, and 9 (adult only) in the posterior tuberculum, and groups 6 (posterior tuberal nucleus), 7, 10, and 11 in the hypothalamus, the latter two detectable in the adult only. The pretectal DA group has also been named group 8. While there are no mesDA neurons in zebrafish, group 1 has been postulated on anatomical basis to represent the TH cells of the retromammillary area, and thus the rostral A9/A10 DA neurons in mammals [28]. These similarities are emphasized by the finding that zebrafish DA neurons of the posterior tuberculum/ventral thalamus form ascending projections into the subpallium, and may thus be involved in neural circuits similar to the mammalian nigrostriatal system [31,32]. These observations are in agreement with the hypothesis that, during evolution, ancient diencephalic equivalents of DA groups A8–A10 might have increased in cell number and expanded laterally and caudally towards mesencephalic territories [33]. Interestingly, in humans the A9, A10 and A11 groups extend along rostrocaudal axis from the midbrain far into the diencephalon, including prosomeres P1 and P2 during development [3,34]. Also, in early mouse development (10–12 days post coitum), mRNA for the rate-limiting enzyme in catecholamine biosynthesis, Tyrosine hydroxylase, is expressed in large continuous rostrocaudal domains extending over the mes-diencephalic border and several forebrain neuromeric domains [2]. This ontogenetic observation is paralleled by an evolutionary perspective which indicates that the mammalian mesDA system may have evolved from a diencephalic ascending DA system from fish through amphibian and birds to mammals [33]. Therefore, it appears likely that early determinants of dopaminergic differentiation may be expressed in similar rostro-caudally extending domains. Here, we investigate in zebrafish the potential contribution of conserved factors to both diencephalic and mesencephalic DA differentiation.

Based on the specific requirement for Nurr1/Nr4a2, Pitx3, and Lmx1b for mesDA development, we decided to address potential functions of these transcription factors for DA neuronal development in zebrafish. Zebrafish Nurr1 homologs [35] have not been studied in detail, but expression of a Medaka fish homolog has been compared with location of DA neurons [36]. Expression and function of zebrafish *lmx1b.1 *and *lmx1b.2 *[37] as well as *pitx3 *[38,39] genes have been studied, but not in relation to DA neurons. Our analyses of co-expression as well as functional knock-down experiments reveal that zebrafish *nurr1/nr4a2 *paralogs are required in a subset of DA precursor cells for control of the neurotransmitter phenotype. Further, *lmx1b *and *pitx3 *are not co-expressed in mature DA neurons of the ventral thalamus and posterior tuberculum. However, *lmx1b *activity appears to be required for specification of these zebrafish DA groups, which likely constitute the fish ascending DA system. Our data suggest that the domain of *lmx1b *expression, extending rostrocaudally from the di- into the mesencephalon in zebrafish, may be an evolutionary ancient domain required for specification of ascending DA systems.

## Results

### Identification of zebrafish Nr4a2/Nurr1 paralogs

Previously, one Nr4a2/Nurr1 homolog was identified during a systematic analysis of zebrafish orphan nuclear receptors [35]. The predicted 41 amino acid peptide (GenBank AAB68748) described in this publication corresponds to the Sanger Ensembl predicted gene ENDSARG00000040850, NCBI Gene 30566. A separate GenScan predicted gene ENDSARG00000017007 exists, which encodes a transcript that contains this peptide in a 584 amino acids ORF with high homology to mammalian Nr4a2, and corresponds to the sequence of the zgc:92696 cDNA [40] located on Chromosome 6 at 11,946 Mb in Assembly Zv6.

In order to identify a potential second Nr4a2 gene resulting from the teleost genome duplication, we searched the Sanger Zv5 Assembly by Blast with the human *Nr4a2 *sequence and identified a 3' fragment of a second *Nr4a2*-like gene. Using RT-PCR as well as Sanger Center EST and genomic trace sequences (see Methods), we sequenced and assembled the complete 598 amino acid ORF of this second *Nr4a2*-like gene. Partial sequence for this second *nr4a2 *gene is contained in ENSEMBL release 44 (Scaffold Zv6_NA1751; ENSDARESTG00000009018), but has not been linked to a chromosome so far.

Sequence alignment of both zebrafish Nr4a2 paralogous proteins with human Nr4a2 revealed 72% amino acid identity for the protein corresponding to the previously published fragment [35], and 88% identity for the second protein predicted from our sequence analysis (Additional files 1 and 2A, B). Based on the higher sequence identity, we name the second zebrafish *nr4a2 *gene, predicted from our sequence to be closer related to the human gene, as *nr4a2a*, while the previously published *nr4a2 *gene is termed *nr4a2b*. Phylogenetic tree analysis reveals that Nr4a2a is the ortholog of the previously published Medaka Nr4a2 [36]. Further, phylogenetic tree analysis with other members of the orphan nuclear receptor family excludes that Nr4a2b is a homolog of other members of the human Nr4a subgroup, including Nr4a1 and Nr4a3 (Additional file 2C, D). Therefore, *nr4a2a *and *nr4a2b *are the duplicated paralogous genes correlating to human *Nurr1/Nr4a2*.

### Analysis of *nr4a2a *and *nr4a2b *expression pattern

**Figure 1:**
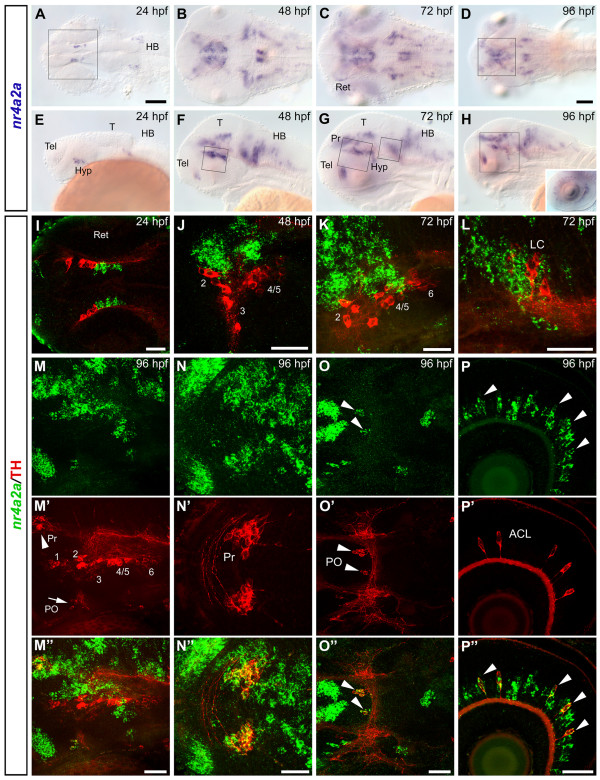
***nr4a2a *is co-expressed with TH in DA neurons of the pretectum, the preoptic area and in amacrine cells of the retina**. (A-H) Whole mount *in situ *hybridization showing *nr4a2a *expression pattern at 24 hpf (A, E), 48 hpf (B, F), 72 hpf (C, G) and 96 hpf (D, H). Dorsal (A-D) and lateral (E-H) views of the head, anterior is to the left. (I-P") The spatial relationship between *nr4a2a*-expressing cells and CA neurons in different areas of the brain was analyzed by whole mount FISH to *nr4a2a *(green) and anti-TH immunohistochemistry (red). Expression was documented by confocal stacks of images, and information for regions corresponding to specific CA neuronal groups was summarized by generation of z-projections from subsets of focal planes of these stacks. (I) Dorsal overview of a 24 hpf embryo (35 μm projection, the approximate head region framed in A is shown): the THir domain is located anterior to the diencephalic *nr4a2a *domain but there is no co-expression. (J) High magnification of the diencephalic DA clusters at 48 hpf (18 μm projection, approximate area framed in F). (K) High magnification of the diencephalic DA clusters at 72 hpf (15 μm projection, approximate area delimited by the big frame in G). (L) High magnification of the region of the locus coeruleus at 72 hpf (6 μm projection, approximate area delimited by the small frame in G). (M-M") Lateral view of a 96 hpf embryo showing the brain region framed in H (23 μm projection): *nr4a2a *(green channel, M) and TH (red channel, M'); co-expression is detectable in the pretectum (Pr, arrowhead in M') and in the preoptic area (PO, arrow in M') (merged channels: M"). High magnification dorsal views of the pretectal area and the preoptic dopaminergic neurons are shown respectively in N-N" (6 μm projection, approximate area framed in D) and in O-O" (11 μm projection, arrowheads). (P-P") *nr4a2a *is expressed in numerous cells of the inner nuclear layer of the retina (P and inset in H), and in all THir amacrine cells (P'-P", arrowheads). In this and in the following figures the DA groups in the ventral diencephalon are numbered from 1 to 6, according to [28]. I, N-N", O-O", dorsal views; J, K, L, M-M", P-P", lateral views; anterior is to the left. Scale bar in A for A-C, E-G and in D for D, H: 100 μm; scale bars in I-P": 50 μm. Abbreviations: **ACL**, amacrine cell layer; **HB**, hindbrain; **Hyp**, hypothalamus; **LC**, locus coeruleus; **PO**, preoptic area; **Pr**, pretectum; **T**, tectum; **Tel**, telencephalon; **Ret**, retina.

**Figure 2:**
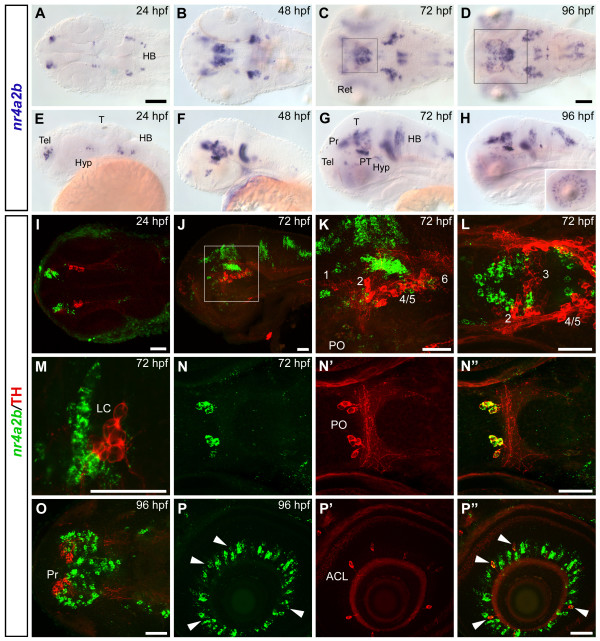
***nr4a2b *is co-expressed with TH in the DA neurons of the preoptic area and in the amacrine cells of the retina**. (A-H) Whole mount *in situ *hybridization showing *nr4a2b *expression pattern at 24 hpf (A, E), 48 hpf (B, F), 72 hpf (C, G) and 96 hpf (D, H). Dorsal (A-D) and lateral (E-H) views of the head are represented, anterior is to the left. (I-P") The spatial relationship between *nr4a2b*-expressing cells and CA neurons in different areas of the brain was analyzed by whole mount FISH to *nr4a2b *(green) and anti-TH immunohistochemistry (red). (I) Dorsal view (56 μm projection) of the head at 24 hpf. (J) Lateral overview (21 μm projection through the diencephalic DA groups) of a 72 hpf embryo. Scattered cells among THir neurons express *nr4a2b *but double labeled cells are not detectable in this region. A higher magnification of the framed area in J is showed in K (15 μm projection), and a dorsal view of the diencephalic clusters at the same developmental stage is presented in L (9 μm projection). (M) Single confocal plane showing the close proximity of *nr4a2b*-expressing cells to the THir NA neurons of the locus coeruleus at 72 hpf. Similar to *nr4a2a*, *nr4a2b *is co-expressed with TH in the preoptic area (N-N", 12 μm projection, 72 hpf) and in the amacrine cells of the retina (arrowheads in P-P", 7 μm projection, 96 hpf), but no co-expression is detectable in the pretectum at 96 hpf (O, 4 μm projection, approximate area framed in D). I, L, M, N-N", O, dorsal views; J, K, P-P", lateral views; anterior is to the left. Scale bar in A for A-C, E-G and in D for D, H: 100 μm; scale bars in I-P": 50 μm. Abbreviations: **ACL**, amacrine cell layer; **HB**, hindbrain; **Hyp**, hypothalamus; **LC**, locus coeruleus; **PO**, preoptic area; **Pr**, pretectum; **PT**, posterior tuberculum; **T**, tectum; **Tel**, telencephalon; **Ret**, retina.

**Figure 3:**
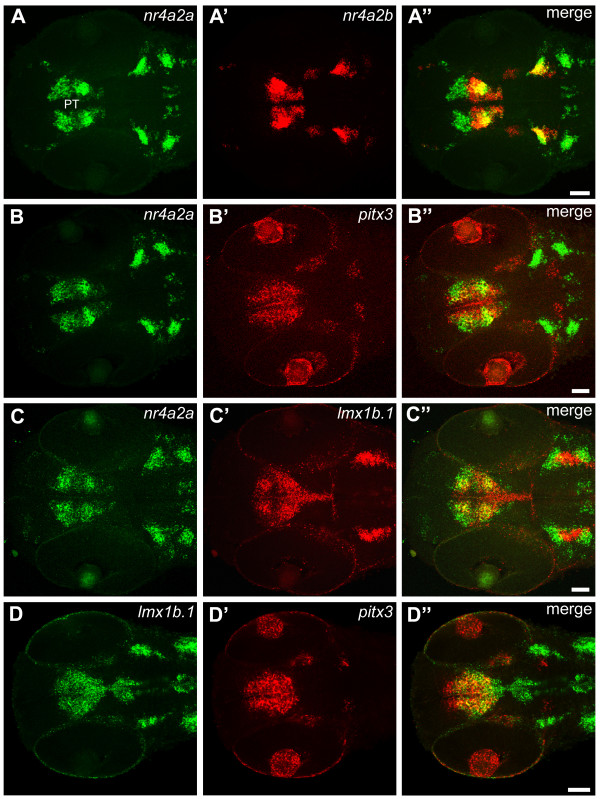
**Areas of co-expression between *nr4a2a/b*, *pitx3 *and *lmx1b.1 *in the posterior tuberculum**. (A-D") Confocal z-projections of double whole mount FISH at 48 hpf show overlapping expression domains for *nr4a2a/b*, *pitx3 *and *lmx1b.1*. The projections encompass the focal planes through the ventral diencephalon. The antisense probes used for the FISH are indicated on the top of each panel. (A-A") 28 μm projection; (B-B") 19 μm projection; (C-C") 16 μm projection; (D-D") 13 μm projection. Anterior is to the left. Scale bars: 50 μm. Abbreviation: **PT**, posterior tuberculum.

We analyzed *nr4a2a *and *nr4a2b *expression patterns by whole mount *in situ *hybridization (WISH) at 24, 48, 72 and 96 hours post fertilization (hpf) (Fig. 1A–H and Fig. 2A–H). Although the expression patterns are similar and overlap largely in most regions of the brain, some differences were observed. At 24 hpf, *nr4a2a *is expressed in few ventral diencephalic cells (Fig. 1A, E) adjacent to a hypothalamic domain defined by *nkx2.1a *expression and posterior of prethalamic *dlx2a *expression (Additional file 3) [41,42]), while *nr4a2b *transcripts first appear in telencephalon and posterior diencephalon (Figs. 2A, E, additional file 3). Both transcripts are expressed in several bilateral cell groups in the anterior hindbrain. At later stages (48–96 hpf; Fig 1B–D, F–H; Fig 2B–D, F–H), the number of *nr4a2a*- and *nr4a2b*-expressing cells increases considerably, and expression domains are more similar albeit not identical. Both transcripts are detected in the ventral telencephalon, in several diencephalic areas (pretectum, thalamus, posterior tuberculum, preoptic area, hypothalamus), in the mid- and hindbrain tegmentum, as well as in the medulla. Moreover, strong expression of *nr4a2a *and *nr4a2b *can be observed in the inner nuclear layer of the retina from 72 hpf onwards (Fig. 1H and 2H insets). To compare precisely similarities and differences in *nr4a2a *and *nr4a2b *expression domains, we have used the Alexa fluorescent dye conjugated Tyramide Signal Amplification (TSA) system to perform double fluorescent *in situ *hybridization. At all examined stages, *nr4a2a *and *nr4a2b *have broad domains of co-expression, but also non-overlapping expression domains in several areas of the brain. In particular, in the posterior tuberculum (Fig. 3A–A"), *nr4a2b *expression is detected only in a posterior subset of cells of the *nr4a2a *expression domain. The observed differences in expression domains are consistent with the hypothesis that the *nr4a2 *genes are the result of a duplication event and may have undergone subfunctionalization, with loss of some promoter elements during evolution.

### *nr4a2a *is co-expressed with TH in dopaminergic neurons of the pretectum, the preoptic area and in amacrine cells of the retina

To be able to determine whether some groups of CA neurons express *nr4a2 *genes, we have combined whole mount fluorescent *in situ *hybridizations with anti-TH immunohistochemistry. At 24 hpf the earliest TH immunoreactive (THir) neurons are located anterior to the diencephalic *nr4a2a *domain. The most posterior THir cells are located in proximity of the anteriormost *nr4a2a*-expressing ones, but there is no co-expression (Fig. 1I). As development proceeds, *nr4a2a *expressing cells are mainly detectable dorsal and medial to the DA neurons of the ventral diencephalon, and are intermingled with THir cells of the more posterior groups, but there is also no co-expression (Fig. 1J, K, M–M"). Similarly, near the locus coeruleus, *nr4a2a*-expressing cells and THir noradrenergic (NA) neurons are in close proximity to each other, but double labelled cells are not observed (Fig. 1L). In contrast, co-expression is detectable in the DA neurons of the pretectum (Fig. 1M–M", N–N") and of the preoptic area (Fig. 1M–M", O–O"), as well as in the DA amacrine cells of the retina (Fig. 1P–P", Table 1). In the telencephalon, at 96 hpf, some scattered *nr4a2a*-expressing cells can be detected around the DA neurons of the subpallial area and of the olfactory bulb, but no co-localization is apparent (Additional file 4A, B).

**Table 1:** Summary of the expression pattern analysis of *nr4a2*, *pitx3 *and *lmx1b *genes relative to each THir cluster at 96 hpf stage.

	**OB**	**SP**	**Pr**	**PO**	**ACL**	**DC – 1**	**DC – 2**	**DC – 3**	**DC – 4**	**DC – 5**	**DC – 6**	**LC**	**MO**
*nr4a2a*	-	-	co-expr.	co-expr.	co-expr.	-	adj	adj	-	adj	-	adj	-
*nr4a2b*	-	-	adj	co-expr.	co-expr.	adj	-	-	adj	-	adj	adj	-
*pitx3*	-	-	-	-	-	adj	adj	-	adj	adj	-	adj	
*lmx1b.1*	-	-	-	-	-	-	adj	-	adj	adj	-	co-expr.	co-expr.
*lmx1b.2*	-	-	-	-	-	-	adj	-	adj	adj	-	adj	adj

'co-expr.' indicates overlapping expression in the considered area, '-' indicates no overlap, 'adj' indicates TH immunoreactivity and gene expression in adjacent cells but no co-expression.

Abbreviations of the different CA groups: **ACL**, amacrine cell layer; **DC-1/6**, diencephalic groups from 1 to 6; **LC**, locus coeruleus; **MO**, medulla oblongata; **OB**, olfactory bulb; **PO**, preoptic area; **Pr**, pretectum; **SP**, subpallium.

### *nr4a2b *is co-expressed with TH in the dopaminergic neurons of the preoptic area and in the amacrine cells of the retina

At 24 hpf, *nr4a2b *transcripts are not detectable in the proximity of THir neurons (Fig. 2I), but at later stages a strong *nr4a2b *domain is observed in the posterior tuberculum, dorsal and medial to the DA neurons. Similar to *nr4a2a*, scattered cells around THir neurons express *nr4a2b*, but co-expressing cells are not detectable in this area (Fig. 2J–L). Co-expression of *nr4a2b *and TH is observed in the preoptic DA cluster (Fig. 2N–N"), and in DA amacrine cells of the retina (Fig. 2P–P", Table 1). In contrast, *nr4a2b*-expressing and THir cells do not overlap in the locus coeruleus and in the pretectum, although they are found in close proximity to each other (Fig. 2M, O). No co-expression of TH and *nr4a2b *is observed in the telencephalon (Additional file 4C, D).

### *pitx3 *and TH are not co-expressed in any catecholaminergic group

**Figure 4:**
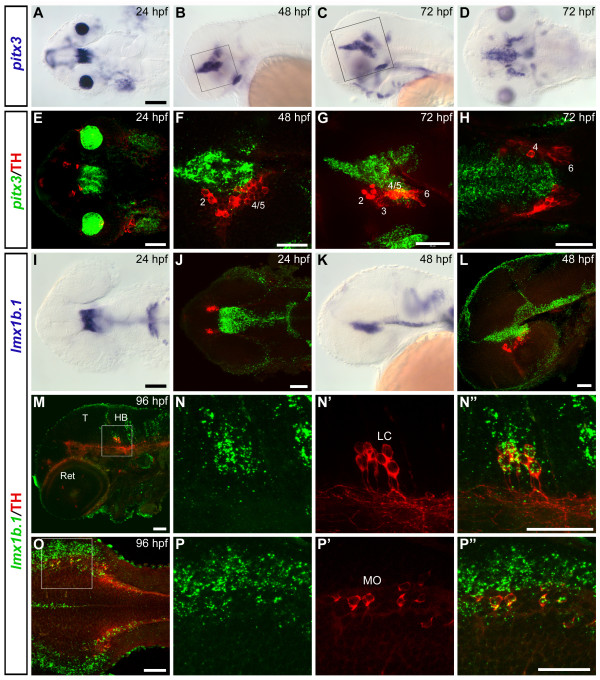
**Expression domains of *pitx3 *and *lmx1b.1 *genes in relation to catecholaminergic groups**. (A-D) Whole mount *in situ *hybridization showing *pitx3 *expression pattern at 24 hpf (A, dorsal view), 48 hpf (B, lateral view) and 72 hpf (C, lateral view, and D, dorsal view). (E-H) Confocal z-projections of whole mount FISH to *pitx3 *(green) and anti-TH immunohistochemistry (red) revealing the spatial relationship between *pitx3*-expressing and THir cells. (E) Dorsal overview of the head at 24 hpf (35 μm projection). (F) Single plane confocal image of a 48 hpf embryo, the approximate diencephalic area framed in B is showed. (G) Lateral view (38 μm projection) of a 72 hpf embryo (area framed in C). (H) Single dorsal plane showing the diencephalic area of a 72 hpf embryo: *pitx3 *expression is mainly detectable in medial and dorsal position with respect to the THir neurons of the posterior tuberculum, and no co-localization can be detected with any DA group (see also additional file 5). (I-L) Expression profile of *lmx1b.1 *at 24 hpf (I-J) and 48 hpf (K-L) analyzed by traditional WISH (I, K) or by whole mount FISH (J, L, green) and anti-TH immunohistochemistry (J, L, red). Dorsal (I-J) and lateral (K-L) views are represented. J and L are 35 μm and 17 μm confocal projections respectively. No co-expression of *lmx1b.1 *and TH is detected in the DA neurons of the posterior tuberculum. Double labelling is instead observed in the NA neurons of the locus coeruleus (LC) (M-N") and medulla oblongata (MO) (O-P"). (M) Single confocal image at the level of the locus coeruleus of a 96 hpf embryo. High magnification of the framed area is showed in N-N" (23 μm projection). (O) Dorsal overview of the medulla oblongata in a 96 hpf embryo (4 μm projection). A high magnification of the framed area is showed in P-P" (single plane) (see also additional files 6 and 7). Anterior is always to the left. Scale bars in A and I (for A-D and I, K): 100 μm; all the other scale bars: 50 μm. Abbreviations: **HB**, hindbrain; **LC**, locus coeruleus; **MO**, medulla oblongata; **T**, tectum; **Ret**, retina.

*pitx3 *is a homeodomain transcription factor that has been recently studied in zebrafish for its role during retinal and pituitary development [38,39,43]. Beside its strong expression in the lens and in the pituitary placode, at 24 hpf *pitx3 *is also strongly expressed in a diencephalic domain posterior to and non overlapping with the earliest THir neurons (Fig. 4A, E). At later stages *pitx3 *diencephalic expression is mainly detectable in positions medial and dorsal to the developing THir neurons of the posterior tuberculum, and no co-expression can be observed with any dopaminergic group (Fig. 4F–H and additional file 5). At 96 hpf, *pitx3*-expressing cells appear even more intermingled with THir neurons, but they are clearly distinguishable from each other and co-expression can be excluded (Additional file 5L, P and Table 1). *pitx3*-expressing cells and the NA neurons of the locus coeruleus are also in close proximity but no overlapping expression is observed (Additional file 5O). No *pitx3 *expression is detected in the telencephalon where the olfactory bulb and the subpallial DA neurons develop.

### *lmx1b.1 *is co-expressed with TH in the noradrenergic neurons of the locus coeruleus and medulla oblongata, whereas *lmx1b.2 *is not expressed in any catecholaminergic group

Lmx1b zebrafish orthologs, *lmx1b.1 *and *lmx1b.2*, have been shown to have similar expression patterns and redundant functions in maintaining the isthmic organizer [37]. Similar to *pitx3*, at 24 hpf both *lmx1b.1 *and *lmx1b.2 *are expressed in a broad diencephalic domain posterior to the earliest THir neurons (Fig. 4I, J and additional file 8A, I). At later stages their expression in the ventral diencephalon is mainly restricted to positions dorsal and medial to the DA neurons (Figs. 4K, L and additional file 6K for *lmx1b.1*, and additional file 8J–L for *lmx1b.2*), and no co-expression of *lmx1b.1 *or *lmx1b.2 *and TH is detected in any DA group of the posterior tuberculum at any examined stage (Additional file 7 and Table 1). In the hindbrain, co-expression of *lmx1b.1 *and TH is observed in the NA neurons of the locus coeruleus (Fig. 4M–N") and medulla oblongata (Fig. 4O–P"), as well as of the area postrema (Additional file 6P–P"). In contrast, although expressed in close proximity, *lmx1b.2 *is not expressed in NA neurons of the hindbrain (Additional file 8M, N).

### *nr4a2a*, but not *nr4a2b*, is required for formation of DA neurons in the preoptic area and the pretectum

**Figure 5:**
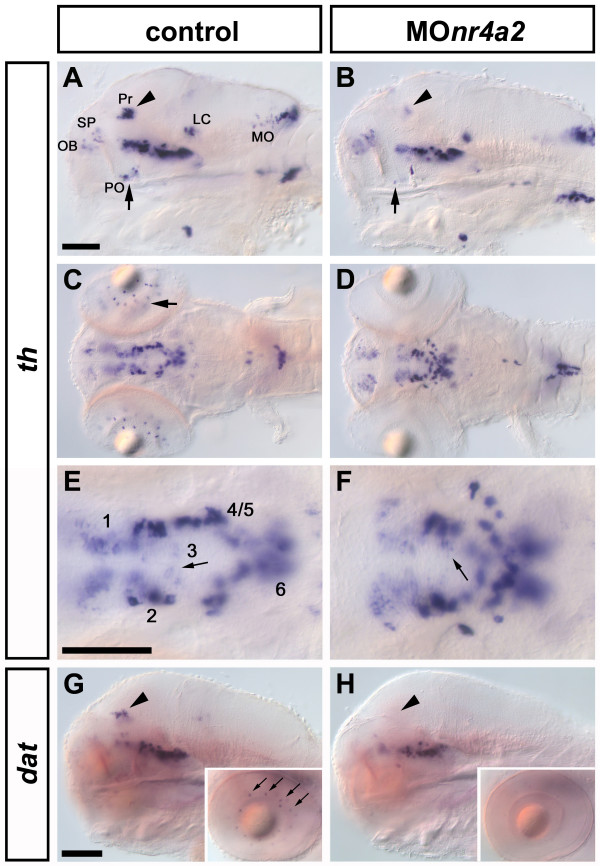
**Nr4a2a is required for formation of DA neurons in pretectum, preoptic area and retina**. Morphants were analyzed by WISH to detect *th *expression at 96hpf. (A) Embryos injected with control morpholino show normal formation of CA groups, including DA nuclei in the pretectum (arrowhead) and the preoptic region (arrow). (B) Injection of 2ng MOnr4a2, which targets both *nr4a2 *genes, leads to a strong reduction of DA neurons in the pretectum (arrowhead) and the preoptic region (arrow). (C) Control morphants form DA amacrine cells in the retina (arrow), which are absent or strongly reduced in embryos injected with MOnr4a2 (D). All the ventral diencephalic DA groups develop in the *nr4a2 *morphant embryos (F), including group 3 (arrow), although the spatial organization of the neurons appears altered when compared to control embryos (E). (G-H) When *dat *expression was analyzed (G, H), *nr4a2 *morphants showed lack of DA neurons in the pretectum (H, arrowhead), in the retina (inset in H) and the preoptic area (not shown). A-B, G-H: lateral views; C-F: dorsal views. Anterior is to the left. Scale bars in A for A-B, in E for E-F and in G for G-H: 100 μm. Abbreviations: **LC**, locus coeruleus; **MO**, medulla oblongata; **OB**, olfactory bulb; **PO**, preoptic area; **Pr**, pretectum; **SP**, subpallium.

We examined by loss-of-function experiments whether *nr4a2a *and *nr4a2b *are involved in catecholaminergic neuron differentiation in the zebrafish. First, we inhibited the translation of both *nr4a2a *and *nr4a2b *by targeting the identical ATG-regions of their mRNAs by morpholino (MO) MOnr4a2. As a control, we used a five mismatch morpholino, mm *nr4a2*-MO, which did not induce any detectable changes when *th *expression pattern was compared with wild-type embryos. MOnr4a2 morphants show complete absence of *th *expression in the preoptic region, the retina and the pretectum by 72hpf (Additional file 9A–D; Table 2). This phenotype is largely maintained at 96hpf, when *nr4a2 *morphants still display a nearly complete absence of *th *expressing neurons in the retina, and either weak or no *th *expression in pretectum and preoptic area (Fig. 5A–D; Table 2). In contrast, the other CA groups develop normally, including the ventral diencephalic DA clusters, although their spatial organization appears slightly altered (Fig. 5E–F).

**Table 2:** Development of CA groups in *nr4a2*, *nr4a2a *and *nr4a2b *morphants.

					**MOnr4a2 + MOp53**	
					
	**MOnr4a2** **72hpf**	**MOnr4a2a** **72hpf**	**MOnr4a2b** **72hpf**	**MOnr4a2** **96hpf**	**72 hpf**	**96 hpf**
**vDC group1**	normal (23/23)	normal (14/17)	normal (25/25)	normal (28/28)*	normal (28/28)	normal (30/30)
**vDC group3**	normal (23/23)	normal (17/17)	normal (25/25)	normal (28/28)	normal (28/28)	normal (30/30)
**posterior vDC**	normal (23/23)	normal (17/17)	normal (25/25)	normal (43/43)	normal (28/28)	normal (30/30)
**PO**	absent (25/26)	absent (16/17)	normal (25/25)	absent (25/43) reduced (15/43)	absent (22/28)	absent (19/30)
**Pr**	absent (25/26)	absent (17/17)	normal (18/25)	absent (10/43) reduced (33/43)	absent (22/28) reduced (6/28)	absent (9/30) reduced (21/30)
**ACL**	absent (25/26)	absent (17/17)	reduced (16/25)	absent (15/43) reduced (28/43)	absent (28/28)	absent (23/30) reduced (7/30)
**OB**	normal (23/23)	n.a.	normal (25/25)	normal (43/43)	normal (28/28)	normal (30/30)
**LC**	normal (26/26)	normal (17/17)	normal (25/25)	normal (43/43)	normal (28/28)	normal (30/30)
**MO**	normal (26/26)	n.a.	reduced (15/25)	normal (43/43)	normal (28/28)	normal (30/30)

The numbers in brackets represent the number of morphants that show the respective phenotypes and the total number of analyzed morphants. (n.a. – not analyzed). Notes: (*) morphants with general morphological defects may sometimes form less DA cells. Abbreviations of the different CA groups: **ACL**, amacrine cell layer; **vDC**, ventral diencephalic; **posterior vDC**, ventral diencephalic groups from 2 and 4 to 6; **LC**, locus coeruleus; **MO**, medulla oblongata; **OB**, olfactory bulb; **PO**, preoptic area; **Pr**, pretectum.

To distinguish between the requirements for *nr4a2a *and *nr4a2b *in preoptic and retinal DA neurons, we next injected morpholinos that specifically inhibit either *nr4a2 *gene. Knock-down of *nr4a2a *completely abolishes *th *expression in the preoptic area, the retina and the pretectum, while all other CA groups develop normally (Additional file 9E–H; Table 2), resembling the phenotype produced by concomitant knock-down of both *nr4a2 *genes. However, *nr4a2b *morphants show normal development of all CA groups, except for a slight reduction of DA amacrine cells (Additional file 9I–L, Table 2).

The absence of *th *expression in pretectum, retina and preoptic area (i.e. in the DA groups that show co-expression of *th *and *nr4a2a*) suggests that Nr4a2a might directly bind to the *th *promoter and regulate its transcription, as it has been shown in rodents [17]. To determine whether other differentiation markers are also absent, we analyzed the expression of *dopamine transporter *(*dat*) [26] in MOnr4a2 injected embryos at 96hpf. When compared to controls, morphant embryos show a strong reduction and/or absence of *dat *expression in pretectum, retina and preoptic area (Fig. 5G, H and data not shown), suggesting that Nr4a2a is necessary for the proper differentiation of these DA neurons. Recently, it has been reported that morpholino injections may induce activation of p53 and apoptosis, and that this effect often is independent of inactivation of the morpholino target gene [44]. Therefore, we controlled experiments by injecting MOnr4a2 together with MOp53, and found the DA phenotype to be identical to MOnr4a2 injection alone. Thus, p53 activation is not involved in loss of DA neurons upon depletion of Nr4a2.

In summary, zebrafish *nr4a2a *is specifically required for development of dopaminergic neurons in the preoptic region, the pretectum and the retina, which co-express *nr4a2a *and *th*. At the same time, there is only a minor requirement for *nr4a2b *during the development of dopaminergic neurons in the retina. While co-expressed in preoptic DA neurons, loss of *nr4a2b *expression may be compensated by *nr4a2a *in preoptic DA cells.

### Knock-down of *pitx3 *does not appear to specifically affect *th *expressing cells

Next, we examined the requirement for *pitx3 *during the development of catecholaminergic neurons in the zebrafish. As reported, *pitx3 *morphants display reduced head and eye size [39], and retinal defects, which were also detected in our experiments (Additional files 10 and 11). Based on the report of extensive apoptosis for *pitx3 *morphant embryos [39], we also co-injected equal amounts of MOp53. While *pitx3 *morphant embryos have severely reduced DA groups, the effect is nearly completely compensated by co-injection of MOp53. We therefore conclude that the DA phenotype observed in *pitx3 *morphant embryos is due to non-specific neural death rather than to a direct involvement of *pitx3 *in the specification or differentiation of DA neurons.

### Knock-down of *lmx1b.1/2 *affects diencephalic DA and hindbrain NA neurons

**Figure 6:**
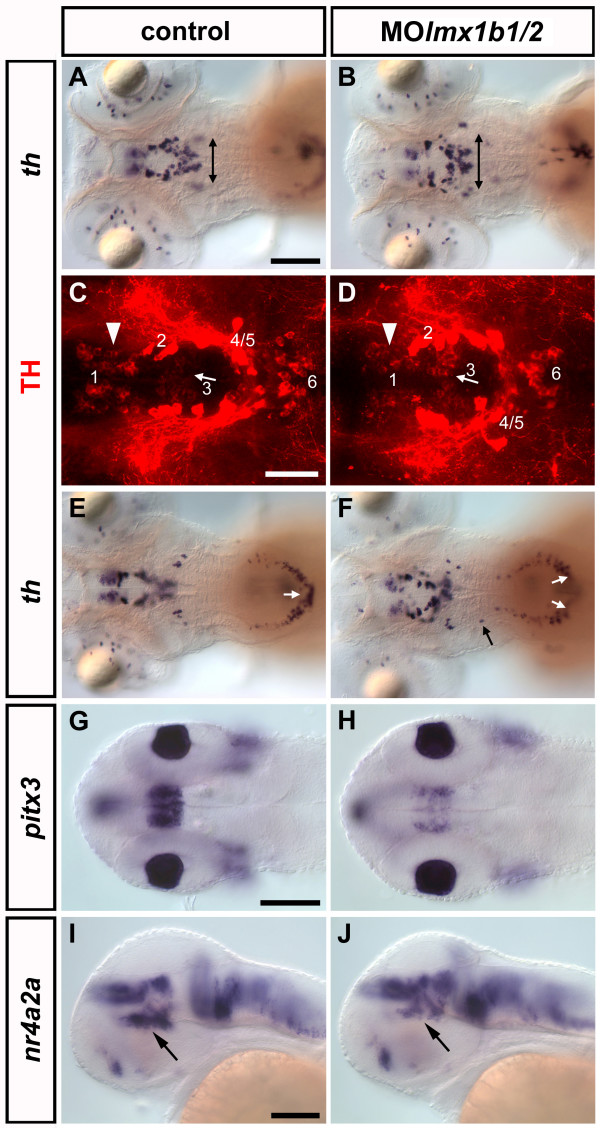
**Loss of *lmx1b.1 *and *lmx1b.2 *changes the spatial organization of CA cell bodies in the vDC and the hindbrain**. (A) Embryos injected with control morpholino display a normal development of DA neurons in the vDC. (B) Embryos injected with both MOlmx1b1 and MOlmx1b2 similarly generate *th *expressing cells in the vDC, but they are dispersed towards lateral positions. (C, D) Confocal analysis of anti-TH immunostaining shows that vDC group 3 neurons develop normally in morphant embryos (D, arrow) compared to controls (C), while group 1 neurons are somewhat reduced (D, arrowhead). Confocal z-projections of 80 μm (C) and 74 μm (D) are reported. (E) Control morphant embryos form noradrenergic neurons in the area postrema (arrow), while (F) in the same area *lmx1b.1 *and *lmx1b.2 *double morphants show a gap of *th *expression (white arrows). Surprisingly, double morphants frequently exhibit ectopic *th*-expressing cells in the hindbrain (F, black arrow). (G-H) Analysis of *pitx3 *expression in control (G) and *lmx1b.1/2 *morphant embryo (H) at 28 hpf. The diencephalic expression domain of *pitx3 *is strongly reduced in MOlmx1b1/2 injected embryos. In addition, the most ventral diencephalic domain of *nr4a2a *expression is reduced in 48hpf morphant embryos (J, compare to I; arrows). A-H: dorsal views; I-J: lateral views. Anterior is to the left. Scale bars in A are for A-B, E-F, in G for G-H and in I are for I-J: 100 μm; scale bar in C is for C-D: 50 μm.

Next, we investigated whether *lmx1b.1 *and *lmx1b.2 *are involved in catecholaminergic neuron development. To this end, we knocked down *lmx1b.1 *and *lmx1b.2 *functions by simultaneous injection of MOlmx1b1 and MOlmx1b2 [37]. At 72 and 96hpf, *lmx1b.1/2 *double morphants display an overall mediolateral broadening of the ventral DC DA groups (Fig. 6A, B and data not shown). The number of dopaminergic cells in DC group 1 is reduced in *lmx1b.1/2 *double morphants as compared to control morphant siblings. Dopaminergic neurons of group 3 and of posterior DC groups form in normal numbers, but the latter are dispersed to more lateral positions (Fig. 6C, D; Table 3). At 96hpf, a fraction of *lmx1b.1/2 *morphant embryos show a reduction of noradrenergic neurons in the area postrema (Table 3). Surprisingly, *lmx1b.1/2 *morphants develop frequently individual ectopic *th*-expressing cells at locations between the LC and the MO (Fig. 6E, F; Table 3). Locus coeruleus neurons, however, appear to form in normal if not slightly increased numbers, but could not be counted accurately due to dense cell clustering (Fig. 6E, F; Table 3).

**Table 3:** Development of CA groups in *lmx1b.1/2 *double morphants.

	**MOlmx1b1/2**		**MOlmx1b1/2 + MOp53**	
	
	**72hpf**	**96hpf**	**72 hpf**	**96 hpf**
**vDC group1**	reduced (36/36)	reduced (71/87)	reduced (10/31)	reduced (13/19)
**vDC group3**	normal (36/36)	normal (87/87)	normal (31/31)	normal (19/19)
**posterior vDC**	dispersed (32/36)	dispersed (72/87)	dispersed (31/31)	dispersed (15/19) reduced (10/19)
**PO**	normal (36/36)	normal (87/87)	normal (31/31)	normal (19/19)
**Pr**	normal (36/36)	normal (87/87)	normal (31/31)	normal (19/19)
**ACL**	reduced (10/36)	normal (87/87)	normal (31/31)	reduced (7/19)
**OB**	reduced (14/36)	reduced(24/87)	normal (31/31)	normal (19/19)
**LC**	n.a.	n.a.	normal (31/31)	normal (19/19)
**MO**	reduced (34/36)	reduced (9/49)	reduced (6/31)	normal (19/19)
**HB ectopic *th***	yes (22/36)	yes (20/49)	yes (5/31)	yes (3/19)

The numbers in brackets represent the number of morphants that show the respective phenotypes and the total number of analyzed morphants. A visual estimate indicated an increase of *th *expressing cells in the LC of *lmx1b.1/2 *morphants, but unambiguous cell counting was not possible due to dense clustering of the cells. Abbreviations are as in Table 2. **HB**, hindbrain.

In order to assess whether the reduction of group 1 DA neurons is specific or rather due to non-specific activation of cell death, we co-injected MOlmx1b1/2 with MOp53 to concomitantly suppress apoptosis [44]. The phenotype of the triple morphant embryos is similar to the one of MOlmx1b1/2 morphants (data not shown and Table 3), indicating a specific involvement of *lmx1b *genes. To test whether the MOlmx1b1/2 morphant DA phenotype might be a late result of early patterning defects, we analyzed expression of diencephalic patterning genes such as *shha *and *dlx2a*, and the differentiation marker *elavl3 *in *lmx1b.1/2 *morphant embryos (data not shown). The results suggest that, in ventral diencephalic areas where DA neurons develop, patterning of the forebrain and the global pattern of neuronal differentiation are normal.

Further, we analyzed *pitx3 *and *nr4a2a *expression in *lmx1b.1/2 *double morphants. At 28hpf, we observe a strong reduction of the diencephalic *pitx3 *expression domain in the morphant embryos compared to controls (Fig. 6G, H). This change in expression level is still detectable at 35hpf, but appears to recover later, as it is no longer detectable at 48hpf (data not shown). Surprisingly, at 48 hpf *lmx1b.1/2 *double morphant embryos show a specific reduction of *nr4a2a *expression in its most ventral diencephalic domain (Fig. 6I, J), while all the other *nr4a2a *domains appear normal. This result suggests that *nr4a2a *expression in the ventral diencephalon might be regulated by Lmx1b activity in their domain of co-expression (see Fig. 3C–C") or alternatively, the reduction of *nr4a2a *expression could be a secondary effect due to the lack of Lmx1b patterning activity in the ventral diencephalon of morphant embryos. In summary, double knock-down of *lmx1b.1 *and *lmx1b.2 *reduces noradrenergic neurons in the area postrema but not in the locus coeruleus, although both regions co-express *lmx1b.1 *and *th*. At the same time, double morphant embryos show a reduction of dopaminergic neurons in DC group 1, despite the fact that the expression domains of *th *and *lmx1b.1 *or *lmx1b.2 *do not overlap in the DC region.

### Lmx1b may define precursor population for DA neurons representing the zebrafish ascending DA system

**Figure 7:**
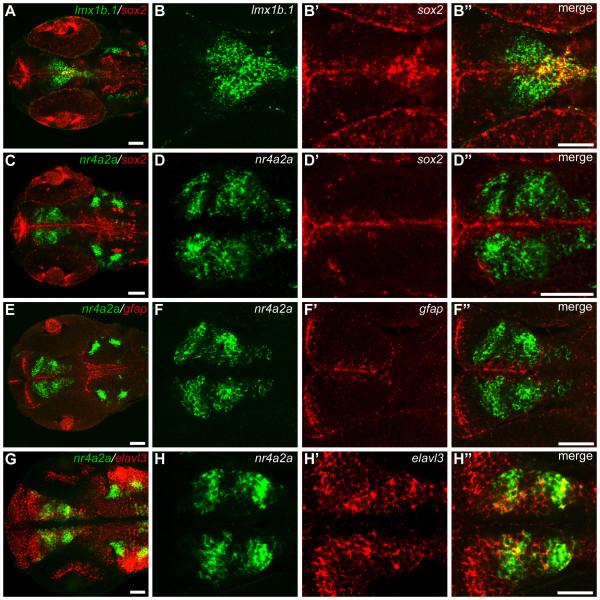
***nr4a2a *is expressed in a population of differentiating neurons**. (A-H") Dorsal views of 48 hpf embryos hybridized with *lmx1b.1 *and *sox2 *(A-B"), *nr4a2a *and *sox2 *(C-D"), *nr4a2a *and *gfap *(E-F"), or *nr4a2a *and *elavl3 *(G-H") probes show that *nr4a2a *is not expressed in progenitor cells, but in early differentiating cells. A, C, E and G show dorsal overviews of the head, at the level of the ventral diencephalon. High magnifications of the posterior tubercular area are reported on the right side of each overview. All the images are single confocal planes, except G, which is a 22 μm confocal projection. Anterior is to the left. Scale bars: 50 μm.

The finding that *lmx1b *genes, although apparently not expressed in mature ventral diencephalic DA neurons, are required for proper formation of DC group 1, suggests that these genes may contribute to the development of DA neuron progenitors. In contrast, the fact that knock-down of *nr4a2 *genes in zebrafish only affects TH expression in neurons that co-express *nr4a2 *indicates that in zebrafish *nr4a2 *genes may be needed for late differentiation of a subset of groups of DA neurons. Therefore, we investigated which of the transcription factors are expressed in neural stem or progenitor cells, mature neurons, or glia. Fluorescent double *in situ *hybridization experiments show that, at 48 hpf, a subset of *lmx1b.1*-expressing cells in the posterior tuberculum co-expresses *sox2*, encoding a transcription factor that at this stage identifies proliferative cells along the ventricle of the diencephalon (Fig. 7A–B"; [45,46]. *nr4a2a*-expressing cells are located 2–3 cell diameter away from the ventricle, and they do not express *sox2 *(Fig. 7C–D"). *gfap*, a marker of proliferative and glial cells, is not expressed in *nr4a2a*-expressing cells (Fig. 7E–F"), while the neuronal differentiation marker *elavl3 *(HuC) is co-expressed at variable levels in *nr4a2a*-expressing cells (Fig. 7G–H"). Analysis at 32 hpf, 36 hpf, and 60 hpf confirmed the results obtained with 48 hpf embryos (data not shown). These results indicate that *nr4a2a*-expressing cells in the posterior tuberculum are differentiating cells of the neuronal lineage.

To investigate the reciprocal spatial relationship of *nr4a2*-, *pitx3*- and *lmx1b*-expressing cells in the diencephalon, we have performed double *in situ *hybridization at 48 hpf. Our results show extensive overlapping expression of *nr4a2a *with both *pitx3 *(Fig. 3B–B") and *lmx1b.1 *(Fig. 3C–C") in the marginal zone of the neuroepithelium, whereas only *pitx3 *and *lmx1b.1 *can be detected along the ventricular zone. However, *pitx3 *and *lmx1b.1 *are co-expressed in both ventricular and marginal cells (Fig. 3D–D"). Altogether, our data show that *pitx3 *and *lmx1b.1 *are expressed in both proliferating and early differentiating progenitor cells of the posterior tuberculum, while *nr4a2a *is expressed in post-mitotic differentiating neurons in the same area.

## Discussion

Although there is considerable mechanistic understanding of midbrain dopaminergic specification in mammals (reviewed by [5,7], it is unclear whether dopaminergic groups in different brain territories are specified by independent developmental pathways, or by shared and evolutionary conserved mechanisms [23,47]. The great evolutionary distance between zebrafish and mammalian systems may be useful in identifying conserved principles of brain development and gene function during neuronal differentiation. To this end, we have analyzed in zebrafish the roles of Lmx1b, Pitx3, and Nurr1/Nr4a2 transcription factors, which have been involved in early neuronal specification and expression of the neurotransmitter phenotype of mammalian DA neurons.

We identified a novel zebrafish *nr4a2 *gene of high sequence similarity to mammalian *nurr1*, which we designate *nr4a2a*, in contrast to the slightly more divergent *nr4a2b *[35]. Analysis of *nr4a2a *expression in relation to dopaminergic neurons was performed using an optimized procedure for fluorescent *in situ *hybridization in combination with anti-TH immunohistochemistry. Analysis of these fluorescent double stains by confocal microscopy enabled us to resolve for individual DA neurons whether they express a specific transcription factor. Our analysis differs from a previous study of *nr4a2 *expression in Medaka [36], which postulated co-expression of *nr4a2 *and *th *in the posterior tuberculum, but may have been unable to distinguish co-expression from expression in adjacent cell layers or in intermingled cells. In zebrafish, we detect *nr4a2a *expression in all pretectal, preoptic, and retinal amacrine DA neurons, but in none of the other DA groups. In contrast, *nr4a2b *was only expressed in preoptic and retinal amacrine DA neurons. Morpholino antisense oligonucleotide based translational knock-down of *nr4a2a *expression revealed that *nr4a2a *is specifically required for expression of *tyrosine hydroxylase *and *dopamine transporter *in all DA neurons that express *nr4a2a*. In contrast, the weak effect of knock-down of *nr4a2b *suggests that it may not be required for DA differentiation, as *nr4a2a *may compensate for the loss of *nr4a2b *function. Simultaneous knock-down of both *nr4a2a *and *nr4a2b *duplicated the *nr4a2a *knock-down phenotype. Our data are consistent with a role of *Nurr1/Nr4a2 *in the control of late differentiation and expression of the DA neurotransmitter phenotype, as suggested for mammalian Nurr1 [14-17,48,49]. However, our data reveal that the subset of DA neurons controlled by *nr4a2 *in zebrafish is different from that in mammals, and DA neurons of the posterior tuberculum/ventral thalamus, which might represent the zebrafish ascending system, do not depend on *nr4a2*, in contrast to mammalian mesDA neurons. Our conclusion is that Nr4a2 is not the sole transcriptional module for DA differentiation. The observation that only a subset of Nr4a2 expressing cells enters DA differentiation indicates that Nr4a2 proteins act in a combinatorial fashion with other transcription factors in late DA differentiation. Our finding that *nr4a2a *is not expressed in *sox2 *expressing stem and progenitor cells, but that all *nr4a2a *expressing cells co-express *elavl3*, a marker for postmitotic differentiating neurons, further supports the notion that Nr4a2 activity contributes to late aspects of dopaminergic differentiation. Finally, as *nr4a2a *is not expressed in *gfap *expressing glia cells, it can be excluded that *nr4a2 *acts in a non-autonomous fashion from adjacent glia cells to support DA differentiation.

We further investigated the contribution to zebrafish DA development of *lmx1b *genes, which have been implicated in early specification of mammalian mesDA progenitors [50], and *pitx3*, which appears to control late aspects of mesDA differentiation [20,51-53]. *lmx1b.1 *does not appear to be expressed in DA neurons, but is co-expressed with TH in NA neurons of the locus coeruleus and the medulla oblongata. *lmx1b.2 *does not appear to be co-expressed with TH in any CA neurons. Interestingly, we find that NA neurons of the area postrema, medulla oblongata, are depleted in *lmx1b.1 *and *lmx1b.2 *combined MO knock-down embryos, while NA neurons of the more rostral medulla and the locus coeruleus develop. While zebrafish *lmx1b *genes are required for maintenance of the mid-hindbrain boundary organizer, *lmx1b *morphants still express organizer signals including FGF8 until about 20 somite stage [37], which appears to be sufficient to pattern the rostral hindbrain and induce locus coeruleus NA differentiation [54]. Although *lmx1b.1 *is co-expressed with *th *in the LC, it is apparently not involved in neurotransmitter specification, similar to its rodent orthologue in the midbrain [50]. Genetically, *lmx1b.1/2 *act upstream of *pitx3 *and *nr4a2 *genes in zebrafish, as Lmx1b function is required to establish normal expression level and domains of *pitx3 *and *nr4a2 *in the ventral diencephalon.

Zebrafish *lmx1b.1 *and *lmx1b.2*, are not co-expressed with any DA neurons in zebrafish. However, surprisingly, morpholino based combined knock-down of *lmx1b.1 *and *lmx1b.2 *reduced the formation of ventral thalamic group 1 dopaminergic neurons. Further, *lmx1b1/2 *knock-down affects both expression of *pitx3 *and *nr4a2 *genes in the ventral diencephalon. As *lmx1b.1/2 *are not expressed in the affected population of differentiated neurons, these findings indicate that it may be required for early specification or maintenance of group 1 DA neurons. As *lmx1b *has been implicated in differentiation and maintenance of DA neurons in the mammalian mesencephalon [14,50,53], we hypothesize that expression of the zebrafish orthologues may mark a progenitor domain of DA neurons. Our findings that *sox2*, a precursor and stem cell marker, is co-expressed with *lmx1b.1*, and that *lmx1b.1 *and *pitx3 *are co-expressed in this precursor territory may support the hypothesis of an evolutionary old *lmx1b *and *pitx3 *expression domain specifying mes-diencephalic DA development. Similar to mouse, not all DA neurons in this domain depend on *lmx1b *genes, and, while a subset of mouse mesDA neurons depends on *pitx3*, such a requirement was not detected in zebrafish. It is interesting to note that in the 2 day old zebrafish embryo, the combined expression domains of *nr4a2a *and *nr4a2b *largely overlap with the expression domains of *lmx1b *genes and *pitx3 *outside the *sox2 *progenitor and stem cell area of the posterior tuberculum and hypothalamus. While genetic analysis in mice indicates that *lmx1b *and *pitx3 *genes contribute to mesDA differentiation independent of *nr4a2 *[50], our analysis in zebrafish indicates that there may be common regulatory mechanisms that define the regions in which all three genes are co-expressed.

## Conclusion

Our data indicate a conserved evolutionary role of Nr4a2 proteins in specification of the DA neurotransmitter phenotype. However, Nr4a2 appears to be only one of several regulatory modules of dopaminergic differentiation, as preoptic, pretectal, and retinal amacrine DA cells depend on Nr4a2, but ventral diencephalic dopaminergic neurons do not express *nr4a2 *genes in zebrafish. In contrast to mammalian Nurr1/Nr4a2 in mesencephalic DA neurons forming ascending projections, Nr4a2 proteins do not contribute to DA neurons of the diencephalic ascending systems in zebrafish. For zebrafish *lmx1b *genes, which are not expressed in mature dopaminergic neurons, our data reveal a role in ventral diencephalic precursor populations contributing group 1 DA neurons. Our data suggest a molecular model to explain the shift and expansion of dopaminergic groups establishing ascending projection systems from the diencephalon into the mesencephalon during evolution from fish to mammals [33]. We hypothesize that rostrocaudal and dorsoventral patterning mechanisms establish a precursor domain that extends from diencephalic prosomere 3 through prosomere 1 into the ventral mesencephalon and defines a competence domain to develop ascending dopaminergic neurons. At the molecular level, this competence domain is established by the co-expression domain of *lmx1b *and *pitx3 *in the posterior tuberculum and dorsal hypothalamus, with *lmx1b *expression extending into the midbrain in fish, but DA development being independent of *pitx3*. During evolution, minor and gradual changes may have enabled an expansion of this competence domain, e.g. posterior expansion of the *pitx3 *expression domain. Such mechanisms may have provided a molecular basis for the rapid shift of ascending DA systems from diencephalon to mesencephalon during vertebrate evolution.

## Methods

### Cloning of *nr4a2a*

TBLASTX of GenBank and Sanger Sequences with human and mouse *Nr4a2 *identified Sanger Centre ENSEMBL scaffold Zv5_NA41, which contained the *nr4a2a *coding sequence, except for a 600 bp gap in the 3' portion of exon 1, as predicted from interspecies comparison. For RT-PCR amplification of this putative 3' fragment of exon 1, we designed a forward primer using the annotated sequence of exon 1 (5'CGCCCTGTCTTTACCAAGCAC3'), and a reverse primer in the annotated sequence of exon 2 (5'CTGACATCTGTTTCTCCGAGG3'). Both primers contain four 3' end mismatches with *nr4a2b*, but only one mismatch with *nr4a2a*, to prevent non-specific amplification of *nr4a2b *sequence. Sequencing and sequence comparison to *nr4a2b *identified a 510 bp fragment as the 3' portion of *nr4a2a *exon 1. The 3' end of exon 1 aligned to a site 10 kb upstream of *nr4a2a *on scaffold Zv5_N41. The assembled 3' flanking sequence was verified by genomic PCR, using a forward primer whose sequence is within the 3' portion of exon 1 (5'GAACCCTGGACAGTCAGAAT3') and a reverse primer containing assembled flanking sequence of the first intron (5'CAGCAGTATTATTATCCCTGAAC3'). The presumptive 30 amino acid gap in the middle of exon 1 was filled by assembly of traces from the genome project (Sanger Institute, UK). Partial *nr4a2a *sequence was found in GenBank XM_001346372 and Ensembl GENSCAN00000001582 on contig Zv6_NA1751, which is not assembled to a chromosome so far. Complete sequence was submitted to GenBank, Accession Number EF661661. Amino acid alignments, calculation of similarity, and phylogenetic tree analysis (Neighbor Joining method of Saitou and Nei) were performed using VectorNTI software.

### Whole-mount in situ hybridization (WISH)

WISH with alkaline phosphatase based color reaction was performed as described [55]. The following digoxigenin-labeled antisense probes were used: *nr4a2a*; *nr4a2b*; *lmx1b.1 *and *lmx1b.2 *[37]; *pitx3 *[38]; *th *and *dat *[26]; *elavl3 *[56]; *sox2 *[46]; *gfap *[57]; *nkx2.1a *[41]; *dlx2a *[42]; *shha *[58].

### Whole-mount fluorescent in situ hybridization (FISH)

Single and double whole-mount FISH were carried out based on reported protocols [59-61] adapted to the zebrafish. Briefly, after rehydration, prior to proteinaseK treatment, the embryos were incubated 30 minutes with 1% H_2_O_2 _in PBT, to quench endogenous peroxidase activity. After hybridization with digoxigenin labeled antisense probe, the embryos were washed as usual and blocked with 1% blocking reagent (Roche #1096176) in TNT buffer (100 mM Tris-HCl pH 7.5, 150 mM NaCl, 0.5% Tween20). To detect the probe we used a 1:400 dilution of anti-DIG antibody POD-conjugated (Roche #1207733). The embryos were then washed several times with TNT buffer and incubated 1 hour with the tyramide-Alexa488 working solution (Molecular Probes, TSA kit #12), protected from light. The tyramide working solution was prepared according to the kit instructions. We have compared the sensitivity of the TSA-Alexa stain to detect gene expression with the standard alkaline phosphatase/BCIP/NBT technique, and found that the TSA-Alexa stain also detects expression weakly stained using alkaline phosphatase and chromogenic substrates (see also Fig. 1, 2 and 4 for comparison). However, we can not exclude that the TSA-Alexa stain misses very low expression levels.

For double FISH, the second antisense probe was always labeled with DNP-11-UTP ribonucleotides (Perkin Elmer, #NEL555). After the first staining step, the tyramide working solution was washed away with TNT buffer and the embryos were incubated 30 minutes with 1% H_2_O_2 _in TNT, to inactivate the peroxidase activity of the first antibody. The embryos were then blocked for 1 hour and incubated overnight with 1:200 dilution of HRP-conjugated anti-DNP antibody (Perkin Elmer, TSA Plus DNP System, NEL747A). The second staining step was performed like the first one, using a different Alexa-conjugated tyramide (Alexa555 or Alexa647, Molecular Probes).

### Immunohistochemistry

After whole mount FISH, a rabbit polyclonal anti-TH primary antibody [24] was used and detected with a goat anti-rabbit Alexa555-conjugated secondary antibody (Molecular Probes). Immunohistochemistry was performed as previously described [62].

### Imaging

Light images were acquired using a Zeiss Axioskop compound microscope equipped with a ProgRes 3008 digital camera. Confocal z-stacks were recorded using Zeiss LSM 510 or a Zeiss LSM 5 DUO laser scanning confocal microscopes.

### Morpholino injections

MOnr4a2 (5'ATACTGAGCCTGGACGCAGGGCATG3') targets the identical 5'-ends of *nr4a2a *and *nr4a2b *mRNAs, at bp -1 to +24. MOnr4a2a (5'CTGAACATGATCTAAAAATACCTTA3') targets the first exon-intron boundary of *nr4a2a*, and MOnr4a2b (5'GTGGTCATTGGCTAATTTTTACCTT3') targets the first exon-intron boundary of *nr4a2b*. MOlmx1b1 and MOlmx1b2 [37] and MOpitx3 [39] target the 5'ends of respective mRNAs. Morpholinos were diluted in 0.05% phenol red or 0.05% rhodamine dextran and H_2_O, and injected into embryos at the 1-cell stage. Injected drop volumes were approximately 2nl, as measured in Halocarbon oil on a micrometer slide. Morpholino concentrations were adjusted to the following amounts per embryo: 2ng MOnr4a2; 6ng MOnr4a2a; 8ng MOnr4a2b; 4ng both MOlmx1b1 and MOlmx1b2; 2–3 ng MOpitx3. Efficient knock-down was confirmed by WISH to *lim3 *in 30hpf *pitx3 *morphants [38], and by WISH to *fgf8 *in 30hpf *lmx1b.1/2 *double morphants [37]. For the *nr4a2 *morpholinos, injection amounts between 0.5 and 8 ng were tested (data not shown), and the lowest concentrations for which *th *expression was efficiently eliminated for DA groups expressing the *nr4a2 *gene was used in subsequent experiments. "Control" embryos were injected with control morpholinos in corresponding quantities: 2ng standard control morpholino (5'CCTCTTACCTCAGTTACAATTTATA3') (GeneTools) as control for MOnr4a2, MOlmx1b1/2 mix and MOpitx3; 6ng, 8ng and 4ng of mismatch MOmcm5 [24] as controls for MOnr4a2a, MOnr4a2b and MOlmx1b1/2 mix, and MOpitx3, respectively. Further, the following mismatch morpholinos were used in same concentrations as the gene specific morpholinos: mm *nr4a2*-MO (5'ATAgTGAcCCTGcACcCAGGcCATG3'); mm *pitx3*-MO1 as published [39]; mm *lmx1bX*-MO as published [37]. MOp53 (5'GCGCCATTGCTTTGCAAGAATTG3') [44] was used in co-injection experiments to assess the specificity of the observed phenotypes. All morpholinos were synthesized by GeneTools (Corvallis, USA).

## Abbreviations

ACL: amacrine cell layer;

CA: catecholaminergic;

DA: dopaminergic;

DC: diencephalic;

FISH: fluorescent in situ hybridization;

HB: hindbrain;

Hyp: hypothalamus;

hpf: hours post fertilization;

LC: locus coeruleus;

mesDA: mesencephalic dopaminergic;

MO: medulla oblongata;

NA: noradrenergic;

OB: olfactory bulb;

PO: preoptic;

Pr: pretectum;

Ret: retina;

SP: subpallium;

T: tectum;

Tel: telencephalon;

TH: Tyrosine hydroxylase;

THir: TH immunoreactive;

vDC: ventral diencephalic;

WISH: whole mount in situ hybridization.

## Authors' contributions

AF performed all gene expression analysis as well as double staining with TH immunohistochemistry, assembled these figures, and wrote the relevant parts of the manuscript. AF also contributed to morpholino knock-down experiments. KD designed and performed the initial set of morpholino knock-down experiments, and contributed to writing. SR contributed the anti TH antibody. MW performed an initial assessment of *nr4a2a *and *nr4a2b *expression domains. JH identified *nr4a2a *sequences from databases. WD conceived the study and participated in the experimental design, performed the cDNA and protein sequence analysis, and prepared the manuscript. All authors read and approved the final manuscript.

## Supplementary Material

Additional file 1 Alignment of zebrafish *nr4a2a *and *nr4a2b *encoded ORFs to Nr4a2 proteins from other vertebrate species. Sequences of Nr4a2 proteins from human (Hs), mouse (Mm), rat (Rn), zebrafish (Dr), medaka (Ol), and pufferfish (Tn) were aligned using VerctorNTi software package. Identical amino acids are highlighted in yellow, highly conserved amino acids in light blue, and conservative amino acid exchanges in green. Click here for file

Additional file 2 Percent identity and dendrograms comparing (A, B) zebrafish Nr4a2 proteins with other vertebrate Nr4a2 proteins, and (C, D) with other members of the orphan nuclear receptor family. Phylogenetic tree analysis of Nr4a2 proteins from human (Hs), mouse (Mm), rat (Rn), zebrafish (Dr), medaka (Ol), and pufferfish (Tn) were performed using VectorNTI software (Neighbor Joining method of Saitou and Nei). Click here for file

Additional file 3 Expression of *nr4a2a *and *nr4a2b *in relation to diencephalic expression domains of *nkx2.1 *and *dlx2a*. Early *nr4a2a *and *nr4a2b *expression domains were analyzed with relation to *dlx2a *and *nkx2.1a *domains by double FISH at 24 hpf (A-B, E-F) and 36 hpf (C-D, G-H). The combination of the different genes is indicated on top of the panels. The images are all confocal z-projections of the planes encompassing *nr4a2a/b *expression in the vDC. At these stages, both *nr4a2a*- and *nr4a2b*-expressing cells are detected posterior to the prethalamic marker *dlx2a *and dorsal to the hypothalamic marker *nkx2.1a*. Anterior is to the left. Scale bars: 50 μm. Click here for file

Additional file 4 *nr4a2 *genes and TH are not co-expressed in the DA neurons of the olfactory bulb and subpallium. (A-D) Confocal z-projections of whole mount FISH to *nr4a2a *(A-B, green) or *nr4a2b *(C-D, green) combined with anti-TH immunohistochemistry (red). Neither *nr4a2a *nor *nr4a2b *is co-expressed with TH in the DA neurons of the subpallial area (A, C) and of the olfactory bulb (B, D). Dorsal views are presented, anterior is to the left. Scale bar: 50 μm. Click here for file

Additional file 5 *pitx3 *and TH are not co-expressed in any CA group. (A-H) Whole mount *in situ *hybridization showing *pitx3 *expression pattern at 24 hpf (A, E), 48 hpf (B, F), 72 hpf (C, G) and 96 hpf (D, H). Dorsal (A-D) and lateral (E-H) views of the head are represented, anterior is to the left. (I-P) Confocal z-projections of whole mount FISH to *pitx3 *(green) and anti-TH immunohistochemistry (red) representing the spatial relationship between *pitx3*-expressing and THir cells. (I) Dorsal overview of the head at 24 hpf (35 μm projection). (J) Single plane confocal image of a 48 hpf embryo, the approximate diencephalic area framed in F is shown. (K) Lateral view (38 μm projection) of a 72 hpf embryo (area framed in G). (L) Lateral view (50 μm projection) of a 96 hpf embryo (frame in H). (M, N) Two different dorsal planes of the same confocal stack (M dorsal to N) showing the diencephalic area of a 72 hpf embryo: *pitx3 *expression is mainly detectable in medial and dorsal position with respect to the THir neurons of the posterior tuberculum, and no co-localization can be detected with any DA group. (O) Dorsal view (15 μm projection) of the locus coeruleus at 96 hpf. (P) Dorsal view (9 μm projection) of the DA groups in the diencephalon at 96 hpf (approximate area framed in D is shown). I, M, N, O, P dorsal views; J, K, L, lateral views; anterior is to the left. Scale bars in A for A-C, E-G and in D for D, H: 100 μm. Scale bars in I-P: 50 μm. Click here for file

Additional file 6 *lmx1b.1 *is co-expressed with TH in the NA neurons of the locus coeruleus and medulla oblongata. (A-H) Whole mount *in situ *hybridization showing *lmx1b.1 *expression pattern at 24 hpf (A, E), 48 hpf (B, F), 72 hpf (C, G) and 96 hpf (D, H). Dorsal (A-D) and lateral (E-H) views of the head are represented, anterior is to the left. (I-P") Confocal z-projections of whole mount FISH to *lmx1b.1 *(green) combined with anti-TH immunohistochemistry (red) representing the spatial relationship between *lmx1b.1*-expressing and THir cells. (I) Dorsal view (35 μm projection) of the head at 24 hpf. (J) Lateral view (17 μm projection) of a 48 hpf embryo. (K) High magnification of the diencephalic area (40 μm projection) in a 96 hpf embryo (approximate area framed in H). No co-expression of *lmx1b.1 *and TH is detected in the DA neurons of the posterior tuberculum. Double labelling is instead observed in the NA neurons of the locus coeruleus (L-M") and medulla oblongata (N-O"), as well as of the area postrema (P-P"). (L) Single confocal image at the level of the locus coeruleus of a 96 hpf embryo. High magnification of the framed area is showed in M-M" (23 μm projection). (N) Dorsal overview of the medulla oblongata in a 96 hpf embryo (4 μm projection). High magnification of the framed area is showed in O-O" (single plane). (P-P") 3 μm dorsal projection through the DA neurons of the area postrema. I, N, O-O" dorsal views; J, K, L, M-M" lateral views; anterior is to the left. Scale bars in A is for A-C, E-G and in D is for D, H: 100 μm. Scale bars in I-P": 50 μm. Click here for file

Additional file 7 *lmx1b.1 *expression in the ventral diencephalon does not overlap with TH in DA neurons. Gallery of 25 confocal images (1 μm apart from each other, from dorsal to ventral) representing the spatial relationship between *lmx1b.1*-expressing (green) and THir (red) cells in the ventral diencephalon. Anterior is to the left. Scale bar: 50 μm. Click here for file

Additional file 8 *lmx1b.2 *is not co-expressed with TH in any CA group. (A-H) Whole mount *in situ *hybridization showing *lmx1b.2 *expression pattern at 24 hpf (A, E), 48 hpf (B, F), 72 hpf (C, G) and 96 hpf (D, H). Dorsal (A-D) and lateral (E-H) views of the head are represented, anterior is to the left. (I-N) Confocal z-projections of whole mount FISH to *lmx1b.2 *(green) followed by anti-TH immunohistochemistry (red) show the spatial relationship between *lmx1b.2*-expressing and THir cells. Although *lmx1b.2*-expressing cells in the diencephalon and in the hindbrain are often intermingled with THir neurons, no co-expression is observed in any of the CA groups. (I) Dorsal overview (32 μm projection) of a 24 hpf embryo. (J) Lateral overview (19 μm projection) of a 48 hpf embryo. (K) Lateral projection (57 μm) of the diencephalic area (framed in G). (L) Lateral projection (56 μm) of the framed region in H. (M) Lateral view (11 μm projection) of the NA neurons in the medulla oblongata at 72 hpf. (N) Dorsal view (6 μm projection) of the locus coeruleus at 96 hpf. Scale bar in A is for A-C, E-G and in D is for D, H: 100 μm. Scale bars in I-N': 50 μm. Click here for file

Additional file 9 Knock-down of *nr4a2a *and *nr4a2b *reveals different requirements for formation of DA neurons in pretectum, preoptic area and retina. Morphant embryos were analyzed for *th *expression by WISH at 72hpf. Embryos injected with control morpholino showed normal formation of CA groups, including DA neurons in the pretectum (A, arrow), the preoptic region (A, arrowhead) and in the retina (C, arrow). The injection of MOnr4a2, which targets both *nr4a2 *genes, leads to a strong reduction of DA neurons in the pretectum (B, arrow), in the preoptic region (C) and in the retina (D). The same phenotype is observed upon injection of a morpholino targeting only *nr4a2a *(MOnr4a2a) (E-F). However, the same DA groups are unaffected by injection of a morpholino targeting *nr4a2b *(MOnr4a2b) (I-L). Knock-down of *nr4a2b *leads only to a mild reduction of *th *expressing DA amacrine cells (L, arrow), as compared to controls (K, arrows). Scale bar: 100 μm. Click here for file

Additional file 10 Knock-down of *pitx3*. *pitx3 *morphants were analyzed at 96hpf for *th *expression by WISH (A-F) and anti-TH immunohistochemistry (G-I), to better visualize groups 1 and 3 in the vDC. (A, D, G) Control embryos show normal development of DA neurons in the retina (A, arrow) and the vDC. (B, E, H) Embryos injected with 2ng MOpitx3 form fewer DA amacrine cells (arrow) and show reduced *th *expression in vDC groups 1 and 3 (E and H, arrowheads point to group 1 and the asterisk to group 3), besides a slight disorganization of the DA groups. Head and eye sizes are decreased as reported by Shi et al. (2005). (C, F, I) Upon co-injection of 2 ng MOpitx3 and 4,5 ng MOp53, the embryos display an almost complete recovery of vDC groups 1 and 3, suggesting that this *pitx3 *morpholino induces off-target effects which lead to an unspecific loss of these groups. A-I: dorsal views. Anterior is to the left. Scale bar in A is for A-C and in D is for D-F: 100 μm; scale bar in G for G-I: 50 μm. Click here for file

Additional file 11 Development of CA groups in *pitx3 *morphants. The numbers in brackets represent the number of morphants that show the respective phenotypes and the total number of analyzed morphants. In MOpitx3 embryos, the posterior vDC DA clusters do form, but cells are often not as tightly organized into clusters as in control embryos (* – in 19/50 morphants, *th *WISH stain intensity appeared stronger in morphants than in controls). CA clusters in the OB and MO are not included here, but may show a slight reduction of *th *expressing cells. Defects observed in MOpitx3 embryos are nearly completely compensated by co-injection of 2 ng MOpitx3 and 4,5 ng MOp53, indicating that the defects are caused by non-specific activation of p53, and that DA neurons may differentiate normally in the absence of Pitx3. Notes: (*) morphants with general morphological defects may sometimes form less DA cells. Abbreviations of the different CA groups: ACL, amacrine cell layer; vDC, ventral diencephalic; posterior vDC, ventral diencephalic groups from 2 and 4 to 6; LC, locus coeruleus; MO, medulla oblongata; OB, olfactory bulb; PO, preoptic area; Pr, pretectum. (n.a. – not analyzed). Click here for file
