# Supplementary figures and images for: Expression and function of nr4a2, lmx1b, and pitx3 in zebrafish dopaminergic and noradrenergic neuronal development
Source: BMC Dev Biol. 2007 Dec 5;7:135. doi: 10.1186/1471-213X-7-135 (PMC2217549; doi:10.1186/1471-213X-7-135)

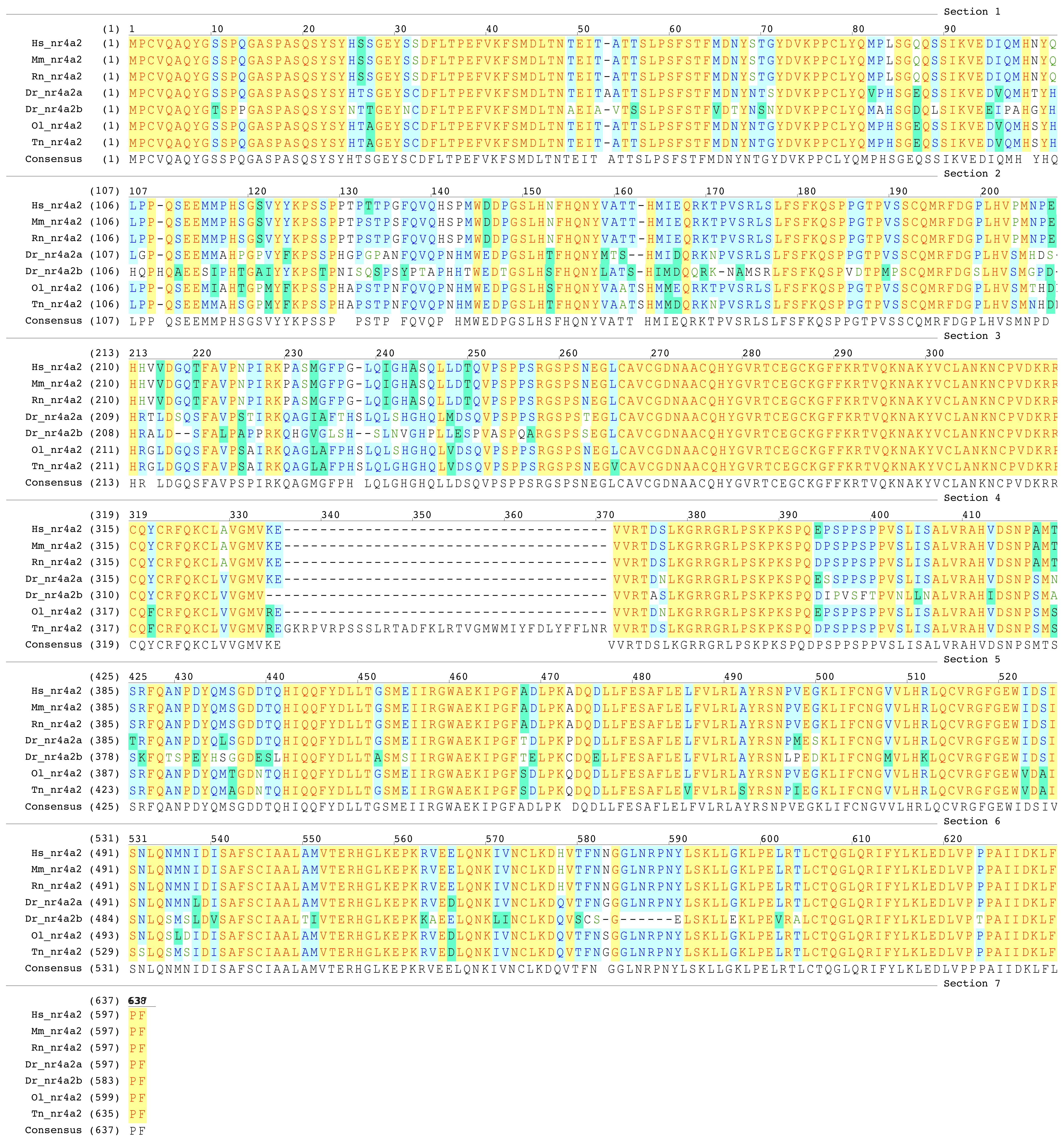

Supplement: Additional file 1 — Alignment of zebrafish nr4a2a and nr4a2b encoded ORFs to Nr4a2 proteins from other vertebrate species. Sequences of Nr4a2 proteins from human (Hs), mouse (Mm), rat (Rn), zebrafish (Dr), medaka (Ol), and pufferfish (Tn) were aligned using VerctorNTi software package. Identical amino acids are highlighted in yellow, highly conserved amino acids in light blue, and conservative amino acid exchanges in green. [file 1471-213X-7-135-S1.jpeg]

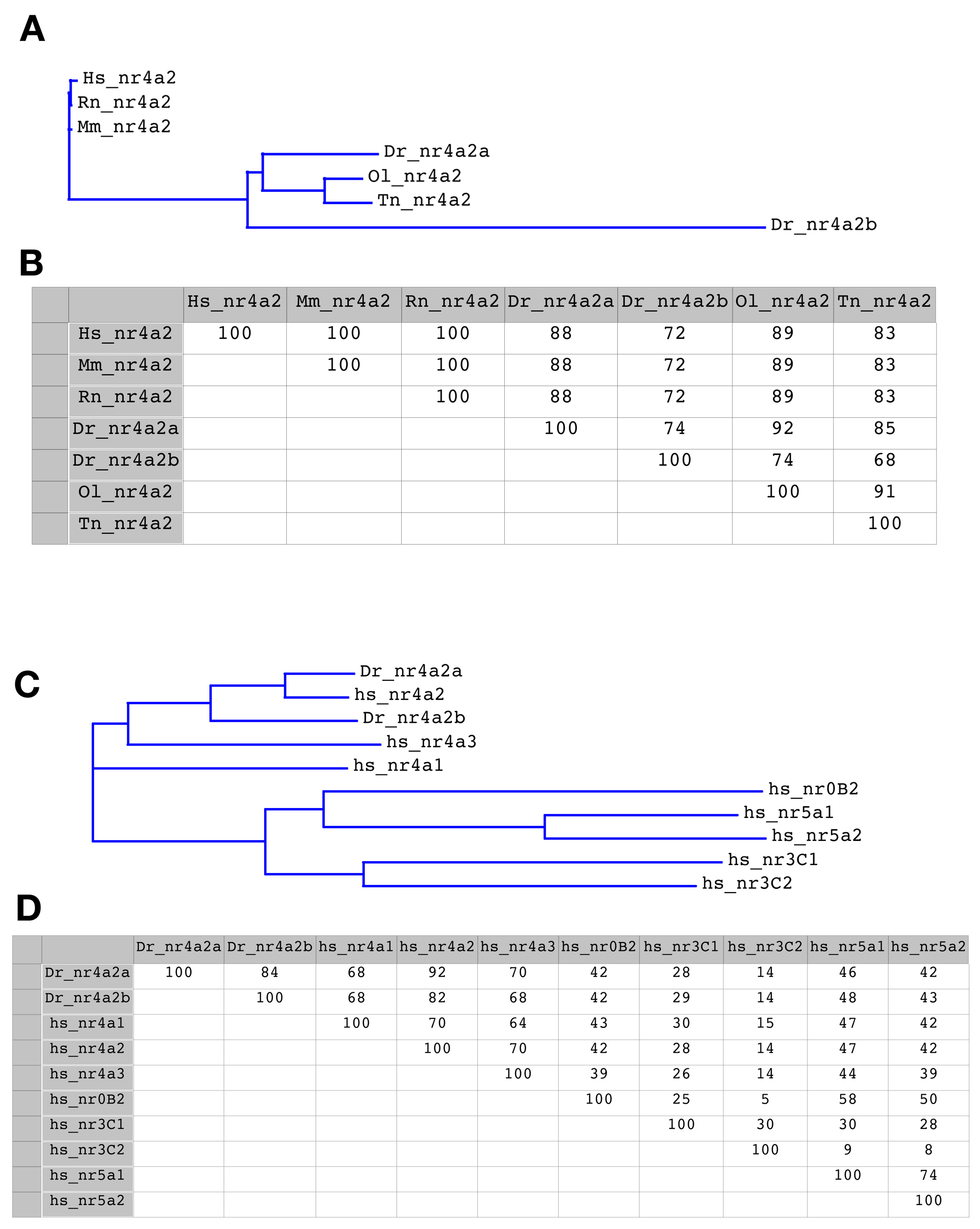

Supplement: Additional file 2 — Percent identity and dendrograms comparing (A, B) zebrafish Nr4a2 proteins with other vertebrate Nr4a2 proteins, and (C, D) with other members of the orphan nuclear receptor family. Phylogenetic tree analysis of Nr4a2 proteins from human (Hs), mouse (Mm), rat (Rn), zebrafish (Dr), medaka (Ol), and pufferfish (Tn) were performed using VectorNTI software (Neighbor Joining method of Saitou and Nei). [file 1471-213X-7-135-S2.jpeg]

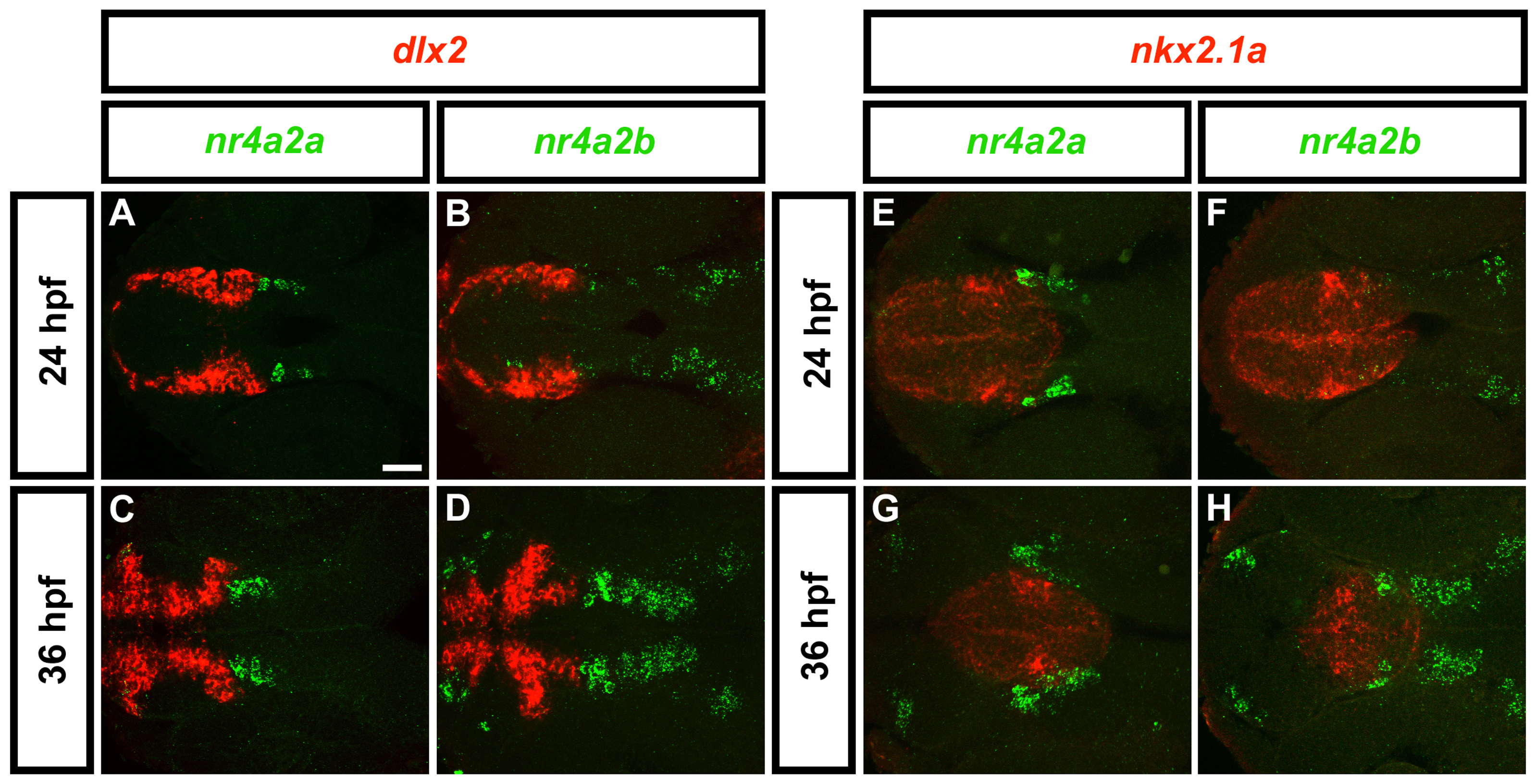

Supplement: Additional file 3 — Expression of nr4a2a and nr4a2b in relation to diencephalic expression domains of nkx2.1 and dlx2a. Early nr4a2a and nr4a2b expression domains were analyzed with relation to dlx2a and nkx2.1a domains by double FISH at 24 hpf (A-B, E-F) and 36 hpf (C-D, G-H). The combination of the different genes is indicated on top of the panels. The images are all confocal z-projections of the planes encompassing nr4a2a/b expression in the vDC. At these stages, both nr4a2a- and nr4a2b-expressing cells are detected posterior to the prethalamic marker dlx2a and dorsal to the hypothalamic marker nkx2.1a. Anterior is to the left. Scale bars: 50 μm. [file 1471-213X-7-135-S3.jpeg]

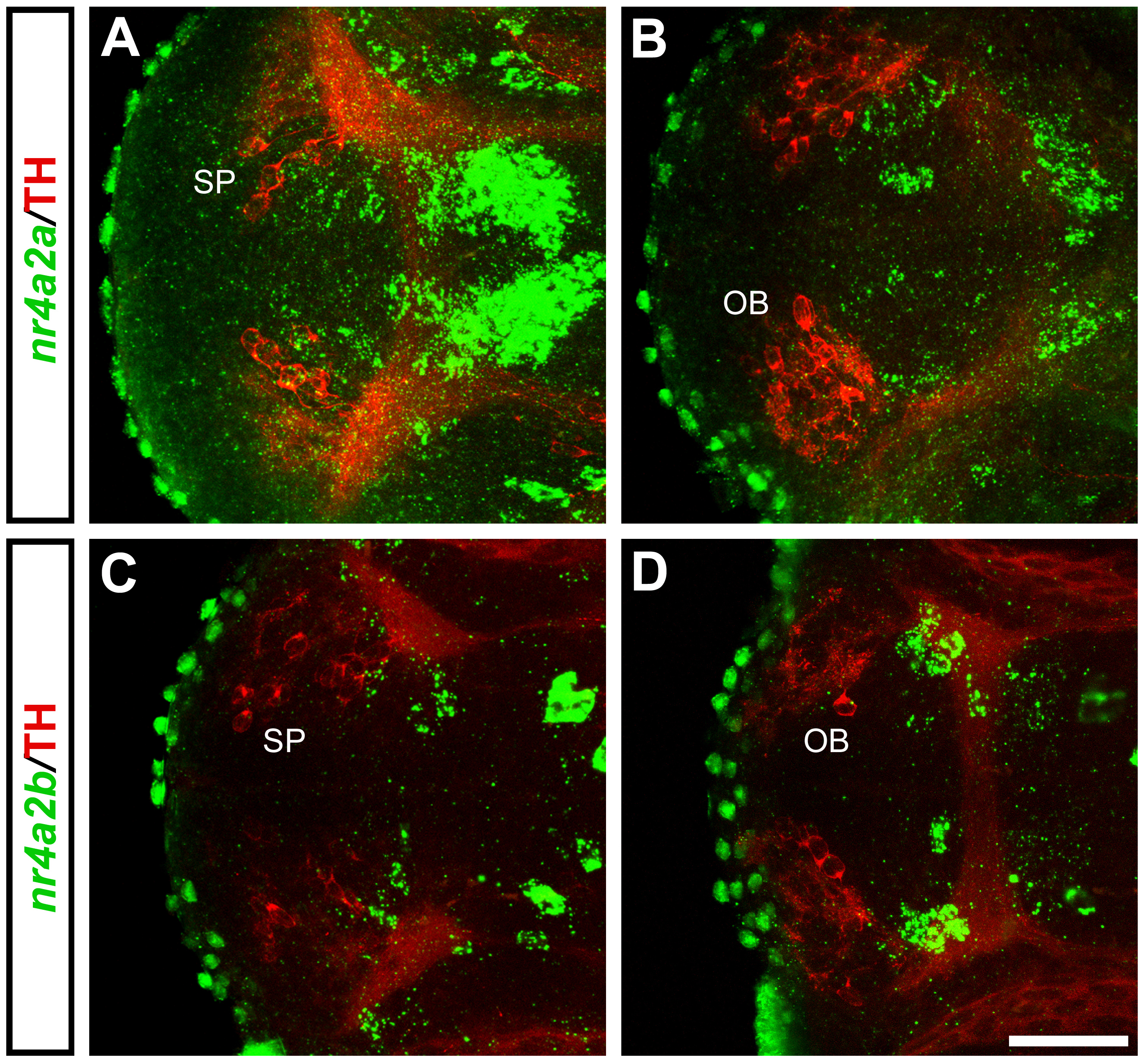

Supplement: Additional file 4 — nr4a2 genes and TH are not co-expressed in the DA neurons of the olfactory bulb and subpallium. (A-D) Confocal z-projections of whole mount FISH to nr4a2a (A-B, green) or nr4a2b (C-D, green) combined with anti-TH immunohistochemistry (red). Neither nr4a2a nor nr4a2b is co-expressed with TH in the DA neurons of the subpallial area (A, C) and of the olfactory bulb (B, D). Dorsal views are presented, anterior is to the left. Scale bar: 50 μm. [file 1471-213X-7-135-S4.jpeg]

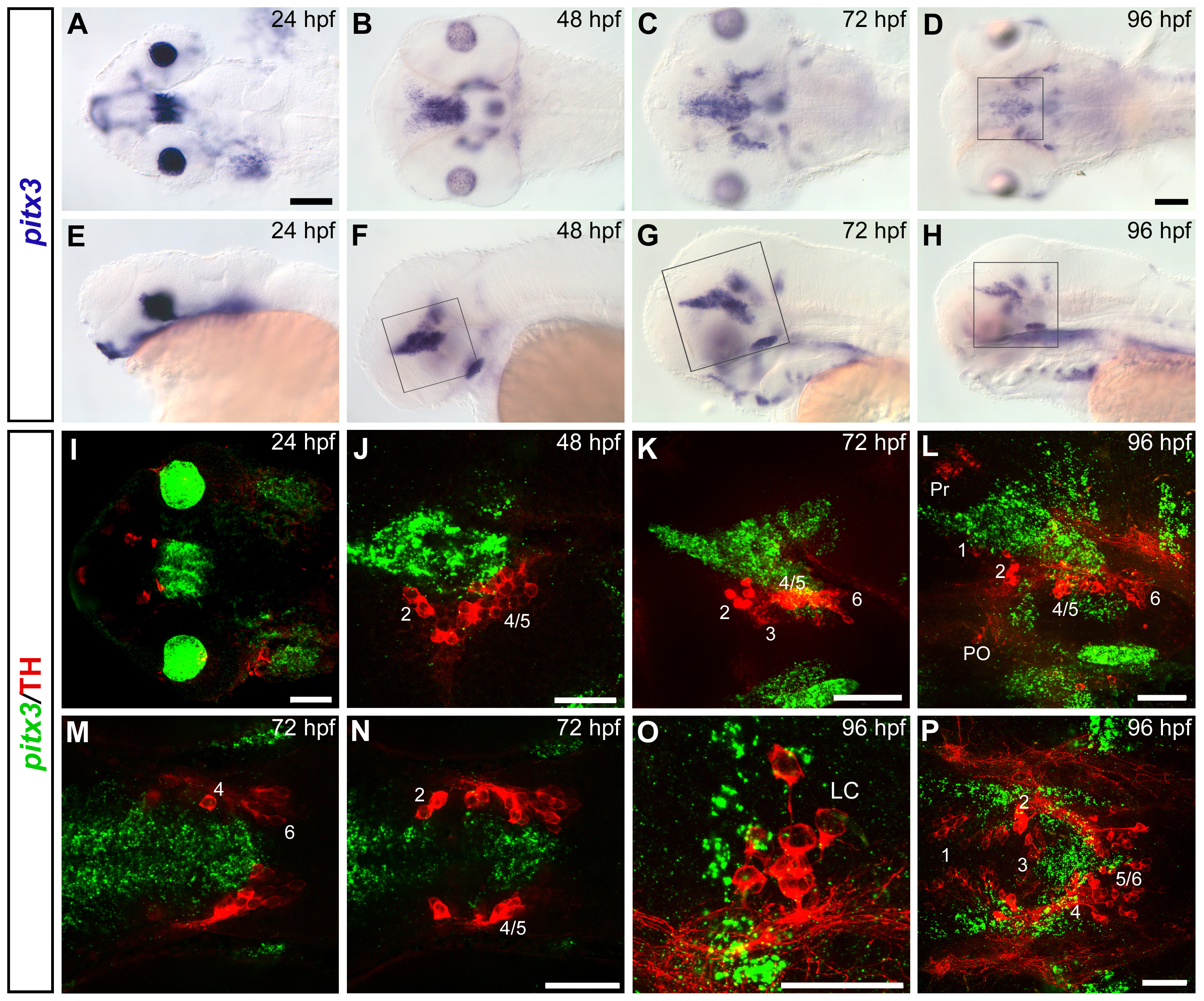

Supplement: Additional file 5 — pitx3 and TH are not co-expressed in any CA group. (A-H) Whole mount in situ hybridization showing pitx3 expression pattern at 24 hpf (A, E), 48 hpf (B, F), 72 hpf (C, G) and 96 hpf (D, H). Dorsal (A-D) and lateral (E-H) views of the head are represented, anterior is to the left. (I-P) Confocal z-projections of whole mount FISH to pitx3 (green) and anti-TH immunohistochemistry (red) representing the spatial relationship between pitx3-expressing and THir cells. (I) Dorsal overview of the head at 24 hpf (35 μm projection). (J) Single plane confocal image of a 48 hpf embryo, the approximate diencephalic area framed in F is shown. (K) Lateral view (38 μm projection) of a 72 hpf embryo (area framed in G). (L) Lateral view (50 μm projection) of a 96 hpf embryo (frame in H). (M, N) Two different dorsal planes of the same confocal stack (M dorsal to N) showing the diencephalic area of a 72 hpf embryo: pitx3 expression is mainly detectable in medial and dorsal position with respect to the THir neurons of the posterior tuberculum, and no co-localization can be detected with any DA group. (O) Dorsal view (15 μm projection) of the locus coeruleus at 96 hpf. (P) Dorsal view (9 μm projection) of the DA groups in the diencephalon at 96 hpf (approximate area framed in D is shown). I, M, N, O, P dorsal views; J, K, L, lateral views; anterior is to the left. Scale bars in A for A-C, E-G and in D for D, H: 100 μm. Scale bars in I-P: 50 μm. [file 1471-213X-7-135-S5.jpeg]

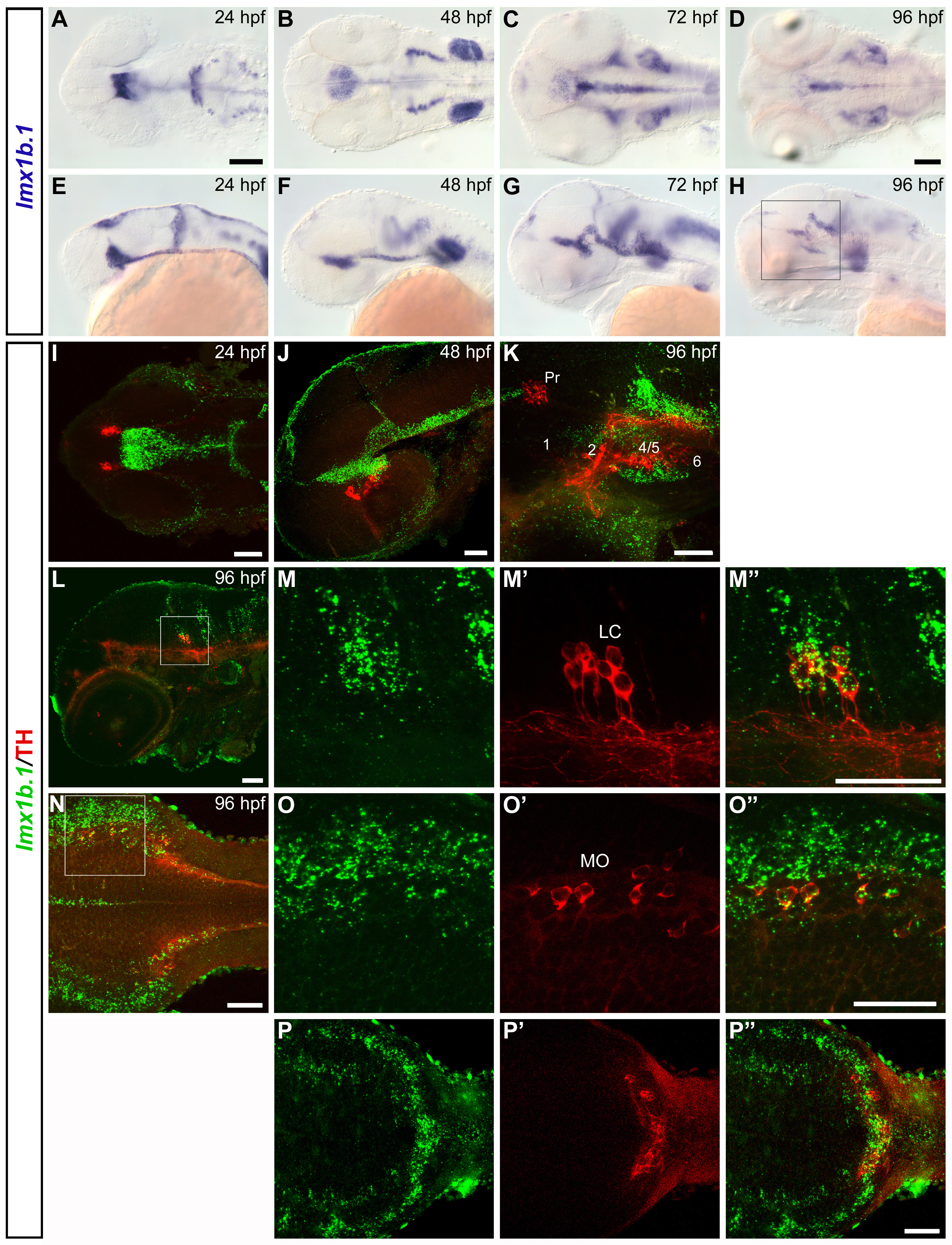

Supplement: Additional file 6 — lmx1b.1 is co-expressed with TH in the NA neurons of the locus coeruleus and medulla oblongata. (A-H) Whole mount in situ hybridization showing lmx1b.1 expression pattern at 24 hpf (A, E), 48 hpf (B, F), 72 hpf (C, G) and 96 hpf (D, H). Dorsal (A-D) and lateral (E-H) views of the head are represented, anterior is to the left. (I-P") Confocal z-projections of whole mount FISH to lmx1b.1 (green) combined with anti-TH immunohistochemistry (red) representing the spatial relationship between lmx1b.1-expressing and THir cells. (I) Dorsal view (35 μm projection) of the head at 24 hpf. (J) Lateral view (17 μm projection) of a 48 hpf embryo. (K) High magnification of the diencephalic area (40 μm projection) in a 96 hpf embryo (approximate area framed in H). No co-expression of lmx1b.1 and TH is detected in the DA neurons of the posterior tuberculum. Double labelling is instead observed in the NA neurons of the locus coeruleus (L-M") and medulla oblongata (N-O"), as well as of the area postrema (P-P"). (L) Single confocal image at the level of the locus coeruleus of a 96 hpf embryo. High magnification of the framed area is showed in M-M" (23 μm projection). (N) Dorsal overview of the medulla oblongata in a 96 hpf embryo (4 μm projection). High magnification of the framed area is showed in O-O" (single plane). (P-P") 3 μm dorsal projection through the DA neurons of the area postrema. I, N, O-O" dorsal views; J, K, L, M-M" lateral views; anterior is to the left. Scale bars in A is for A-C, E-G and in D is for D, H: 100 μm. Scale bars in I-P": 50 μm. [file 1471-213X-7-135-S6.jpeg]

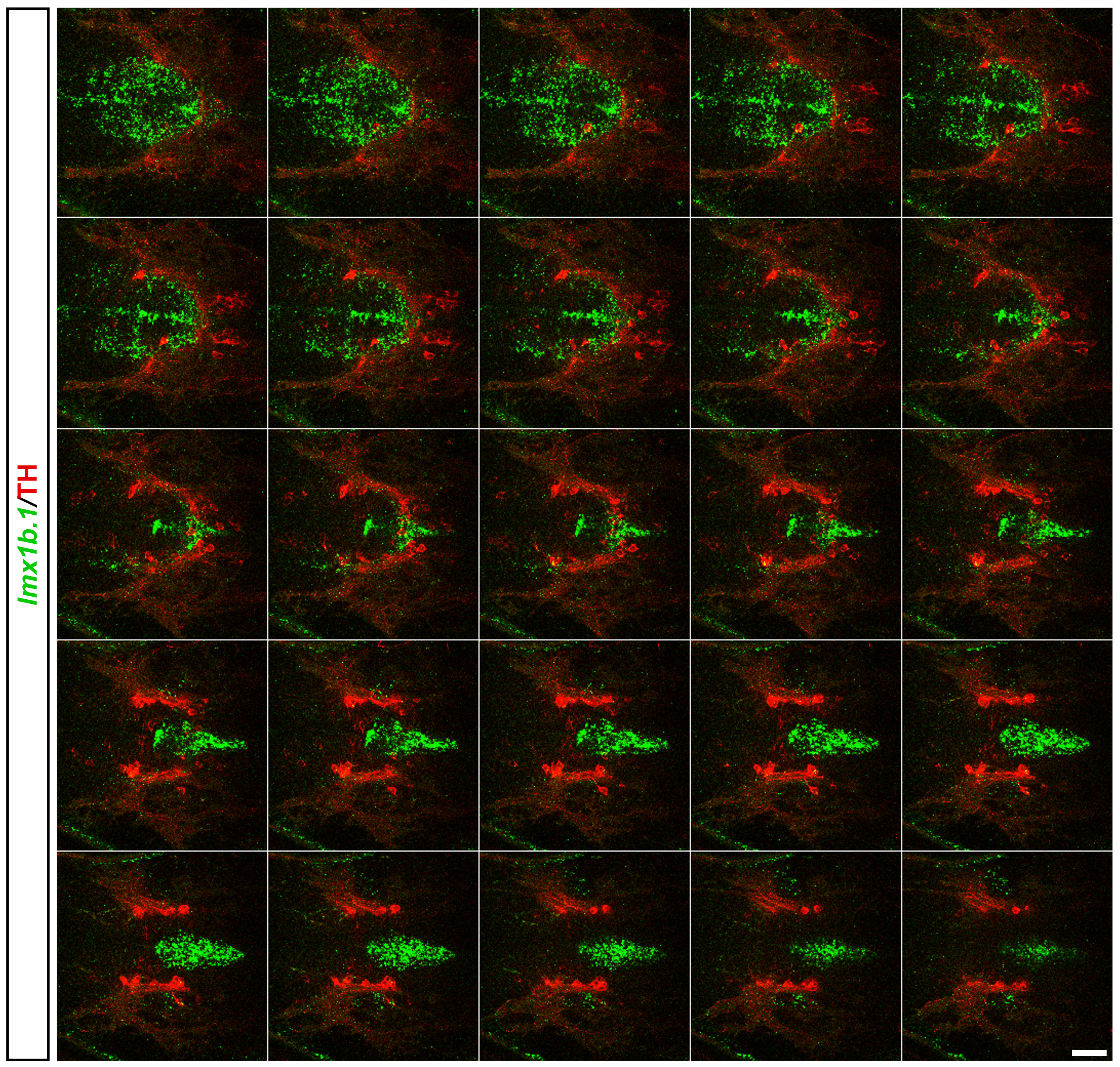

Supplement: Additional file 7 — lmx1b.1 expression in the ventral diencephalon does not overlap with TH in DA neurons. Gallery of 25 confocal images (1 μm apart from each other, from dorsal to ventral) representing the spatial relationship between lmx1b.1-expressing (green) and THir (red) cells in the ventral diencephalon. Anterior is to the left. Scale bar: 50 μm. [file 1471-213X-7-135-S7.jpeg]

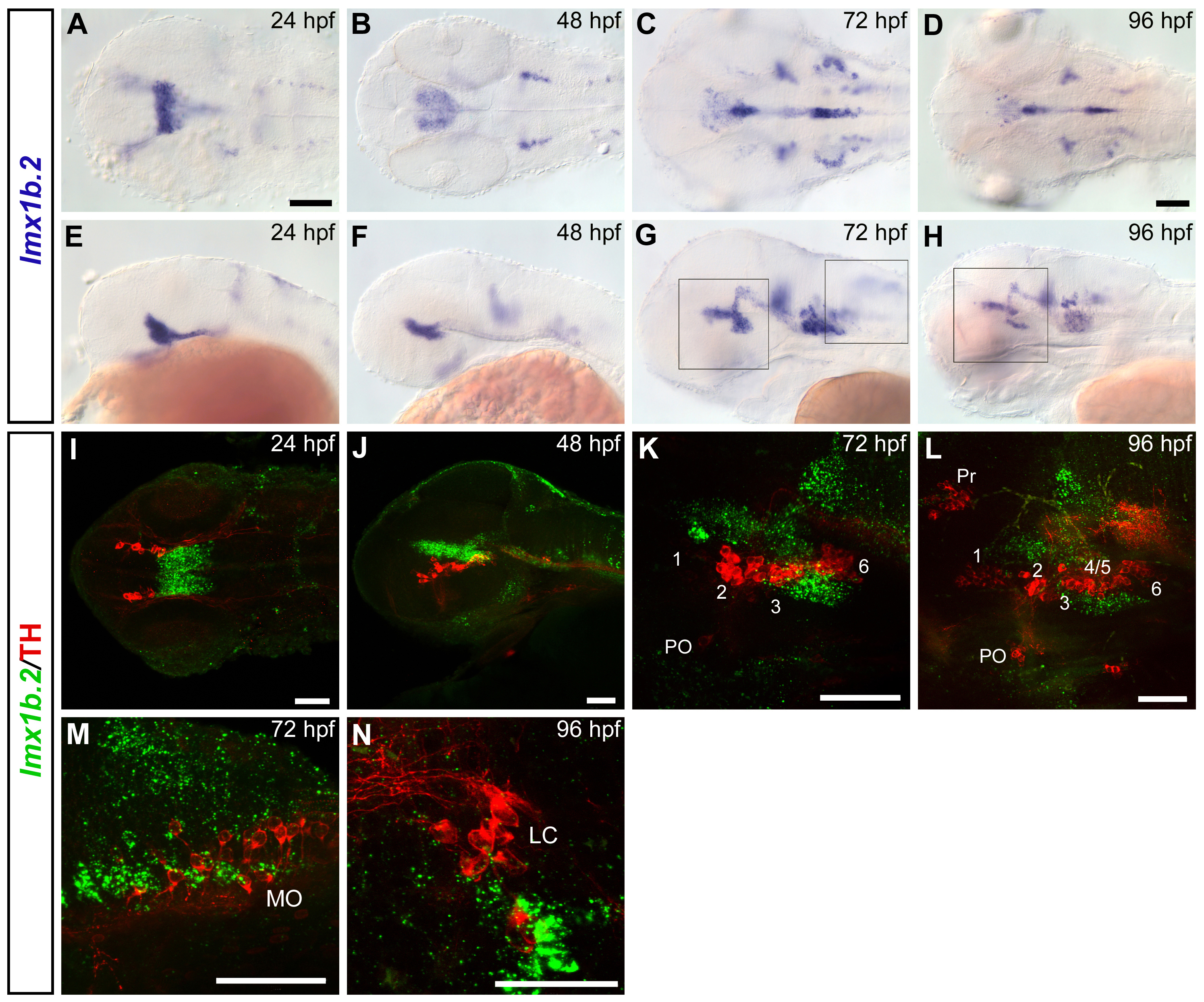

Supplement: Additional file 8 — lmx1b.2 is not co-expressed with TH in any CA group. (A-H) Whole mount in situ hybridization showing lmx1b.2 expression pattern at 24 hpf (A, E), 48 hpf (B, F), 72 hpf (C, G) and 96 hpf (D, H). Dorsal (A-D) and lateral (E-H) views of the head are represented, anterior is to the left. (I-N) Confocal z-projections of whole mount FISH to lmx1b.2 (green) followed by anti-TH immunohistochemistry (red) show the spatial relationship between lmx1b.2-expressing and THir cells. Although lmx1b.2-expressing cells in the diencephalon and in the hindbrain are often intermingled with THir neurons, no co-expression is observed in any of the CA groups. (I) Dorsal overview (32 μm projection) of a 24 hpf embryo. (J) Lateral overview (19 μm projection) of a 48 hpf embryo. (K) Lateral projection (57 μm) of the diencephalic area (framed in G). (L) Lateral projection (56 μm) of the framed region in H. (M) Lateral view (11 μm projection) of the NA neurons in the medulla oblongata at 72 hpf. (N) Dorsal view (6 μm projection) of the locus coeruleus at 96 hpf. Scale bar in A is for A-C, E-G and in D is for D, H: 100 μm. Scale bars in I-N': 50 μm. [file 1471-213X-7-135-S8.jpeg]

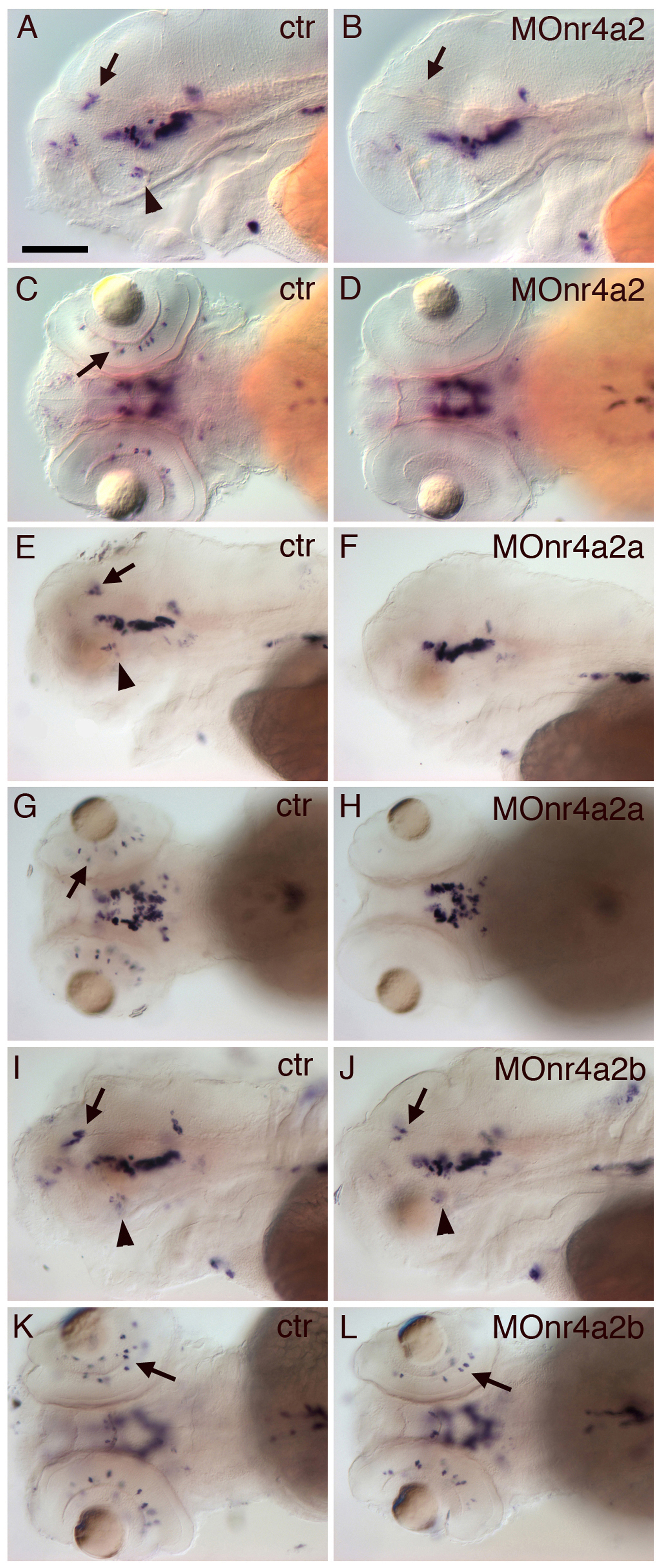

Supplement: Additional file 9 — Knock-down of nr4a2a and nr4a2b reveals different requirements for formation of DA neurons in pretectum, preoptic area and retina. Morphant embryos were analyzed for th expression by WISH at 72hpf. Embryos injected with control morpholino showed normal formation of CA groups, including DA neurons in the pretectum (A, arrow), the preoptic region (A, arrowhead) and in the retina (C, arrow). The injection of MOnr4a2, which targets both nr4a2 genes, leads to a strong reduction of DA neurons in the pretectum (B, arrow), in the preoptic region (C) and in the retina (D). The same phenotype is observed upon injection of a morpholino targeting only nr4a2a (MOnr4a2a) (E-F). However, the same DA groups are unaffected by injection of a morpholino targeting nr4a2b (MOnr4a2b) (I-L). Knock-down of nr4a2b leads only to a mild reduction of th expressing DA amacrine cells (L, arrow), as compared to controls (K, arrows). Scale bar: 100 μm. [file 1471-213X-7-135-S9.jpeg]

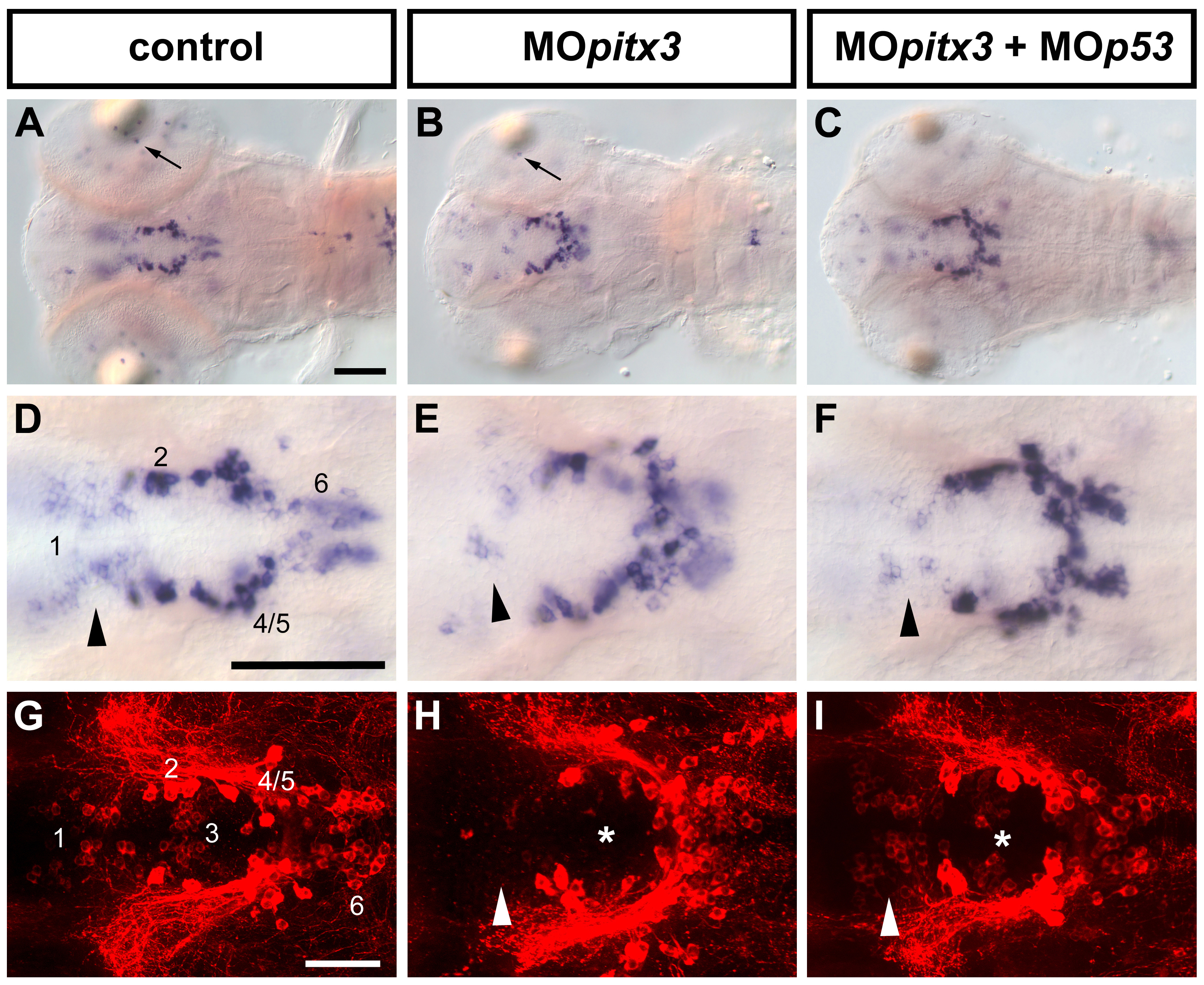

Supplement: Additional file 10 — Knock-down of pitx3. pitx3 morphants were analyzed at 96hpf for th expression by WISH (A-F) and anti-TH immunohistochemistry (G-I), to better visualize groups 1 and 3 in the vDC. (A, D, G) Control embryos show normal development of DA neurons in the retina (A, arrow) and the vDC. (B, E, H) Embryos injected with 2ng MOpitx3 form fewer DA amacrine cells (arrow) and show reduced th expression in vDC groups 1 and 3 (E and H, arrowheads point to group 1 and the asterisk to group 3), besides a slight disorganization of the DA groups. Head and eye sizes are decreased as reported by Shi et al. (2005). (C, F, I) Upon co-injection of 2 ng MOpitx3 and 4,5 ng MOp53, the embryos display an almost complete recovery of vDC groups 1 and 3, suggesting that this pitx3 morpholino induces off-target effects which lead to an unspecific loss of these groups. A-I: dorsal views. Anterior is to the left. Scale bar in A is for A-C and in D is for D-F: 100 μm; scale bar in G for G-I: 50 μm. [file 1471-213X-7-135-S10.jpeg]
