# Supplementary material for: Expression and function of nr4a2, lmx1b, and pitx3 in zebrafish dopaminergic and noradrenergic neuronal development
Source: BMC Dev Biol. 2007 Dec 5;7:135. doi: 10.1186/1471-213X-7-135 (PMC2217549; doi:10.1186/1471-213X-7-135)
Supplement: Additional file 11 — Development of CA groups in pitx3 morphants. The numbers in brackets represent the number of morphants that show the respective phenotypes and the total number of analyzed morphants. In MOpitx3 embryos, the posterior vDC DA clusters do form, but cells are often not as tightly organized into clusters as in control embryos (* – in 19/50 morphants, th WISH stain intensity appeared stronger in morphants than in controls). CA clusters in the OB and MO are not included here, but may show a slight reduction of th expressing cells. Defects observed in MOpitx3 embryos are nearly completely compensated by co-injection of 2 ng MOpitx3 and 4,5 ng MOp53, indicating that the defects are caused by non-specific activation of p53, and that DA neurons may differentiate normally in the absence of Pitx3. Notes: (*) morphants with general morphological defects may sometimes form less DA cells. Abbreviations of the different CA groups: ACL, amacrine cell layer; vDC, ventral diencephalic; posterior vDC, ventral diencephalic groups from 2 and 4 to 6; LC, locus coeruleus; MO, medulla oblongata; OB, olfactory bulb; PO, preoptic area; Pr, pretectum. (n.a. – not analyzed). [file 1471-213X-7-135-S11.doc]

**Additional File 11**

**Title: Development of CA groups in *pitx3* morphants.**

|  | MOpitx3 | | MOpitx3 + MOp53 | |
| --- | --- | --- | --- | --- |
|  | **72hpf** | **96hpf** | **72 hpf** | **96 hpf** |
| **vDC group1** | absent (24/24) | reduced (36/48) | reduced (8/40)  normal (32/40) | reduced (6/36)  normal (30/36) |
| **vDC group3** | absent (22/24) | reduced (16/18) | n.a. | n.a. |
| **posterior vDC** | cluster organization abnormal  (20/24) | cluster organization abnormal  (38/50) * | normal 40/40 | normal (36/36) |
| **PO** | normal (14/20) | n.a. | normal (40/40) | normal (36/36) |
| **Pr** | absent (34/34) | reduced (58/97) | normal (40/40) | normal (36/36) |
| **ACL** | absent (33/34) | reduced (68/70) | absent (9/40)  reduced (7/40) | reduced (21/36) |
| **LC** | reduced (24/34) | normal (79/97) | normal (40/40) | normal (36/36) |
